# Charge-Based Separation of Micro- and Nanoparticles

**DOI:** 10.3390/mi11111014

**Published:** 2020-11-18

**Authors:** Bao D. Ho, Jason P. Beech, Jonas O. Tegenfeldt

**Affiliations:** Division of Solid State Physics and NanoLund, Physics Department, Lund University, P.O. Box 118, 22100 Lund, Sweden; bao.hodang@gmail.com (B.D.H.); jason.beech@ftf.lth.se (J.P.B.)

**Keywords:** electrokinetic deterministic lateral displacement, charge-based separation

## Abstract

Deterministic Lateral Displacement (DLD) is a label-free particle sorting method that separates by size continuously and with high resolution. By combining DLD with electric fields (eDLD), we show separation of a variety of nano and micro-sized particles primarily by their zeta potential. Zeta potential is an indicator of electrokinetic charge—the charge corresponding to the electric field at the shear plane—an important property of micro- and nanoparticles in colloidal or separation science. We also demonstrate proof of principle of separation of nanoscale liposomes of different lipid compositions, with strong relevance for biomedicine. We perform careful characterization of relevant experimental conditions necessary to obtain adequate sorting of different particle types. By choosing a combination of frequency and amplitude, sorting can be made sensitive to the particle subgroup of interest. The enhanced displacement effect due to electrokinetics is found to be significant at low frequency and for particles with high zeta potential. The effect appears to scale with the square of the voltage, suggesting that it is associated with either non-linear electrokinetics or dielectrophoresis (DEP). However, since we observe large changes in separation behavior over the frequency range at which DEP forces are expected to remain constant, DEP can be ruled out.

## 1. Introduction

Deterministic Lateral Displacement (DLD) is a powerful size-based particle sorting method that has multiple advantages: It is label-free, it has high resolution, and it allows for continuous operation [1]. The method has been applied to sort various types of cells or bio-particles from blood: white blood cells [2,3,4,5,6], circulating tumor cells [7,8,9,10,11,12], trypanosomes [13,14], nucleated red blood cells [15], microvesicles [16,17], and PC3 prostate cancer cells [18]. In addition, DLD has found applications in separation or enrichment of different types of mammalian cells [19,20,21,22,23], fungal spores [24], droplets [25,26], bacteria [27], DNA [1,28], and exosomes [29]. Extensive reviews of DLD can be found elsewhere [30,31]. Here we give a brief description of the method.

**Figure 1:**
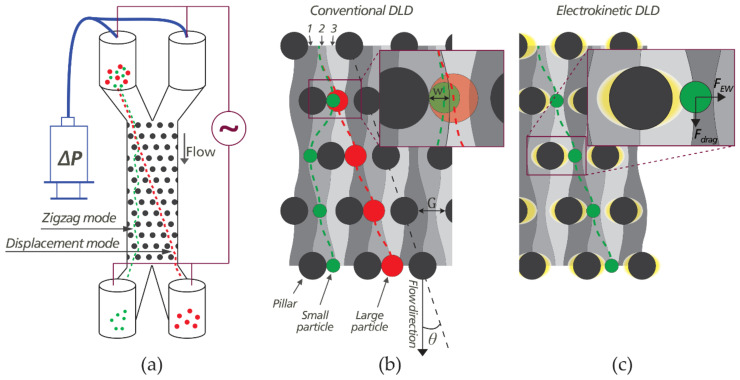
Working principle of conventional Deterministic Lateral Displacement (DLD) and DLD with electric fields (eDLD). (**a**) Diagram of a typical DLD device. The device is driven by a pressure difference between inlets and outlets and is capable of sorting a mixture of particles by size. Electrokinetic effects are added by applying a voltage along the device. In this case, the average field is parallel to the flow. (**b**) Mechanism of conventional DLD. Separation is a result of consecutive migration of large particles across different flow streams (here numbered 1, 2, 3) due to steric interactions with the pillars. (**c**) In eDLD, the particle trajectories are modified by electrokinetic wall force fields around the pillars.

A schematic of a DLD device is shown in Figure 1a. The main feature is an array of micro-pillars that is tilted at an angle with respect to the flow carrying the particles. A heterogeneous sample containing small and large particles is loaded into the sample reservoir on the top left of the schematic in Figure 1a and driven through the pillar array. The DLD mechanism is fundamentally based on whether or not particles are able to follow the carrier fluid as they moves through the array. At low Reynolds numbers it is primarily steric interactions between the particles and the posts that prevent this but under certain conditions other interactions play a role such as charge interactions when the carrier fluid has low ionic strength [32] or inertia and wall lift forces when Reynolds numbers are high [33]. These additional interactions can be carefully chosen to be sensitive to relevant particle parameters and we will show here how externally applied AC fields make it possible to sort particles by their zeta potential in a tunable manner.

Unlike sieves, which also hinder particles from following the flow, steric and other interactions lead to changes in trajectories only and DLD devices can therefore be considered as non-clogging sieves. The DLD mechanism causes different-sized particles to follow different trajectories so that they can be collected in separate reservoirs at the end of the device.

The mechanism of conventional DLD (steric interactions only) is illustrated in Figure 1b. The pillar array repeats itself after *N* rows where *N* is called the period of the DLD. Due to the specific arrangement of the pillar array, the fluid flow is split into *N* different streams between any two neighboring pillars in each row. For a device with equal lateral and longitudinal inter-post distance, the corresponding tilting angle of the array is given by *tan(θ)* = 1/*N*. For the sake of convenience and to avoid anomalous modes [34], the design is often such that *N* is an integer. As an example, in Figure 1b, the period is *N* = 3, corresponding to a tilt of *arctan*(1/3) = 18.4°.

The separate trajectories of one small particle (green) and one large particle (red) are illustrated in Figure 1b. The radius of the green particle is smaller than the stream width *w* depicted in Figure 1b, and as a result, the particle’s center of mass remains in stream #2 throughout the array. This mode of transport is termed the zigzag mode due to the shape of the trajectories. In contrast, the red particle whose radius is larger than *w* bumps into the pillar and is displaced from stream #2 to stream #3. This stream-switching occurs every time the particle encounters a new row of pillars, forcing the particle to travel along the tilting angle of the array. This mode is termed the *displacement mode* and we will refer to the angle *θ* henceforth as the *displacement angle*. At the end of one period (*N* rows) of the pillar array, particles in zigzag mode have a maximum lateral displacement of *G + D,* where *G* is the gap between posts and *D* is the post diameter, while particles in displacement mode are shifted laterally by a distance *N × (G + D)*. Using the angular formulation instead, the displacement of the smaller particles is ≈0 and that of the large particles is *l × tan(θ)*, where *l* is the length of one period (*N* rows) of the pillar array. The *critical diameter D_C_*, which is the diameter at which the transition between zigzagging and displacing occurs, is predetermined by the geometry of the array, and can be estimated by:

(1) $$D_{C}=1.4GN^{-0.48}$$

Equation (1), an empirical formula reported by Davis [35], is used throughout this paper to estimate the critical diameters of our DLD devices.

Although the behaviors of particles in DLD devices are primarily dependent on their size, size is not always a well-defined property of a particle. Instead, we use the term effective size to relate the particle’s trajectory to the trajectory of a perfectly spherical particle moving through a DLD device driven solely by a pressure driven flow. In other words, an arbitrary particle under arbitrary experimental conditions is said to have an effective size *D_eff_* if it moves along the same trajectory as a perfectly spherical particle of size *D_particle_* = *D_eff_* experiencing only the pressure driven flow and steric interactions in the DLD array. This is useful to describe, for example, non-spherical, deformable, or charged particles that have effective sizes that depend on the detailed properties of the particles along with the exact experimental conditions being used. Previous approaches have targeted one or more of these properties to separate particles in DLD based on, for example, shape [13,14,36,37], length [27], and deformability [6,36,38]. Surface charge has also been employed in the context of DLD [32,39], but in general in a different manner than in our current work. Zeming et al. [32] modulated the effective size by tuning the electrostatic force between pillars and nanoparticles, which was achieved by adjusting the salt concentration of the running medium. The method works well for particles below 1 µm in low-salt medium (≤1 mM NaCl). In contrast, our approach, with the integration of an electric field, can be used for micro as well as nano particles, and operates at higher salt concentrations that can be relevant for biological samples.

The interaction between particles and applied electric fields is complicated. A full description must take into account the behavior of the solvent (water) and the dissolved ions near the particle surface. The zeta potential of a particle is simply the electrostatic potential at the shear plane due to the charges on the particle. The zeta potential depends on the density of charged groups on the surface of the particle, the distance between the particle surface and the shear plane, and the ionic strength of the suspending medium. Because the zeta potential is both easy to measure (taking several minutes with a Zetasizer NanoZS instrument (see Section 2.1) and captures the behavior of the effective charge of the particle as the ionic strength of the buffer changes, we will use “zeta potential” throughout and not “charge”, but the reader should keep in mind that they are related.

The use of electric fields for particle manipulation in a liquid medium encompasses multiple mechanisms, among which electrophoresis and dielectrophoresis are the most notable. Electrophoresis (EP) refers to the movement of charged particles in an electric field, which can be uniform or non-uniform [40,41,42,43]. It should be noted that EP of charged particles is in fact “force-free”: the particle motion is driven by the electroosmotic slip on the particle surface. The applications of electrophoresis to manipulate and separate particles and molecules are countless, and it has become a gold standard for separation of proteins and nucleic acids. Dielectrophoresis (DEP) refers to the movement of polarizable particles (charged or uncharged) in a polarizable medium under a non-uniform electric field [44,45,46]. Although having a shorter history than electrophoresis, DEP has also found numerous applications in enrichment, isolation, or separation of bio-particles, including yeast cells [47,48,49,50], human cells [51], microalgae [52], bacteria [53,54,55], viruses [56], DNA [57,58], proteins [59], and nanocrystals [60], to name but a few. Dielectrophoretic techniques can be categorized into Microelectrode-based DEP (eDEP) or Insulator-based DEP (iDEP) [61]. As their names suggest, these two branches are distinguished by the materials the particles are in contact with: conducting electrodes (eDEP) or insulating obstacles (iDEP). For eDEP systems, in the early days, the electrodes were pin-and-plate macroelectrodes [47] but using microfabrication, electrodes have been miniaturized to achieve higher field strengths. Various configurations of electrodes have been reported in the literature, with castellated [48,49,56,62,63], quadrupole polynomial [56,64], zipper [65], or 3D-well [66] designs. In iDEP systems, efforts are invested in designing innovative insulating structures inside microfluidic channels to achieve the desired field gradients for DEP. These structures include constrictions [51,59,60,67,68,69], ridges [70,71], curves [50,72], and obstacle arrays [53,57,73,74,75]. A thorough review of iDEP can be found elsewhere [61].

In terms of configuration, the electrokinetic DLD devices presented in this paper are relatively similar to an iDEP system with an insulating pillar array. The key difference is, however, that our devices have pillar arrays tilted at an angle to the flow to achieve the DLD function. Furthermore, we are interested in not only DEP, but also other electrokinetic phenomena that enhance particle displacement. In general, any electrokinetic force fields that are specific to some particle property and that change the probability that particles switch streams at every row of pillars (altering the cumulative lateral displacement of the particles), can be exploited for tunable particle separations (Figure 1c). The nature of different electrokinetic forces will be discussed in Section 3.4.

Some examples of the applications of electric fields in DLD (eDLD) have been reported. In the first DLD paper [1], a DC electric field was used to transport DNA through a DLD device via electrophoresis and separation of kilo base pair DNA was achieved, but here the electrophoresis was seen solely as a means to drive the DNA through the array. Hanasoge et al. also used DC field to drive the flow and explore the effect of different array orientations on particle separation in eDLD [76]. The development of eDLD has been driven primarily by the Tegenfeldt group at Lund University, Sweden, [77,78,79] and Morgan group at University of Southampton, UK, [39,80,81], who have made use of AC fields in various modes and geometries. Among the benefits of using AC fields are the fact that the flow (carrying the particle through the device) is decoupled from the forces near to the posts (causing separation) and that much greater control can be achieved through the balancing of these forces. What is more, the nature of the force and the property of the particle that is targeted can be controlled via the frequency of the applied electric field.

In the first eDLD devices reported by Beech et al. [77], macro electrodes located inside the inlet and outlet reservoirs were used to apply an AC electric field parallel to the direction of the pressure-driven flow. The effective critical sizes were tuned in a controlled manner by up to a factor of four, for the applied voltages used. Subsequently, higher electric fields have been achieved by having electrodes right on top of an open pillar array [78] and more recently by coating pillars with platinum (3D electrode) [79] and applying the voltage between the rows of pillars. The novel 3D electrode approach obtains the closest possible inter-electrode distances, increasing the field strength per applied voltage by about three orders of magnitude. This active pillar eDLD device is able to displace particles with diameters of 250 nm, much smaller than the gap size of 11 µm. In a different approach, the Morgan group increased the field strength in eDLD by integrating microelectrodes on the sides of the pillar array. Using this configuration, Calero et al. demonstrated sorting of 500 nm from 1 and 3 µm polystyrene beads in a DLD array with a critical size of 6.3 µm using an AC voltage (50 Hz–10 kHz) [80]. In their subsequent work, by using an AC voltage with a DC bias, they were able to separate 100 nm from 500 nm and 1 µm polystyrene beads as well as sorting based on zeta potential for 3 µm microspheres [39]. Finally, in a very recent paper, Calero et al. compared their experimental data at 50 kHz with DEP numerical simulation and found good agreement [81].

Here we expand on our previous work with eDLD, using the simplest approach, i.e., macro electrodes located inside inlet and outlet reservoirs, and show tunable separation based on zeta potential of a variety of particles. We characterize and optimize our devices with regard to different running parameters using polystyrene beads with diameters from several micrometers down to 100 nanometer scales and with a wide range of zeta potentials. Finally, we apply the technique to the sorting of bio-relevant particles: Nanoscale liposomes with different sizes and zeta potentials. Liposomes have found tremendous applications in medicine and pharmacology [82] since their inception in the mid-1960s, most notably for drug delivery. What is more, liposomes are structurally and chemically similar to extracellular vesicles (EVs)—cell-derived particles involved in intercellular communication, and as such are highly relevant as a model system.

Nanoscale bio-particles, such as liposomes or EVs [83,84,85,86], have considerable potential in diagnostic and therapeutic applications. However, sorting of nanoscale particles has been identified as an important challenge [87]. Sorting of EVs down to 20 nm has been demonstrated [29] using nanofluidic DLD with feature sizes on the scale of 100 nm, but the fluidics is difficult to control and the risk of clogging is high. In addition, fabrication of these devices requires sophisticated and expensive processes like electron beam lithography, limiting broad usage. Conversely, devices with features on the micrometer scale can be fabricated using commonly available methods. We show that eDLD devices with micrometer scale gaps can be an alternative approach to the separation of nanoparticles, without the scaling down of device features and thus without some of the problems in fabrication and operation that such scaling down entails.

To optimize eDLD for different applications, it is necessary to understand how different parameters individually or mutually affect the separation. For example, increasing driving pressure can improve throughput (particle numbers or volumes), which is of great interest in many applications, but it also means a higher voltage is required to effectively displace particles. The maximum voltage that can be applied is limited by electrolysis and possibly by Joule heating through the device geometry and the conductivity of running medium. However, some particle systems place constraints on the conductivities of the suspending medium that can be used. To untwine complex dependencies as such, we scan and investigate the influence of medium conductivity, voltage, frequency, and pressure on the efficiency of separation and establish a few simple rules that may help employing eDLD for biological applications.

## 2. Materials and Methods

### 2.1. Devices and Experimental Setup

**Figure 2:**
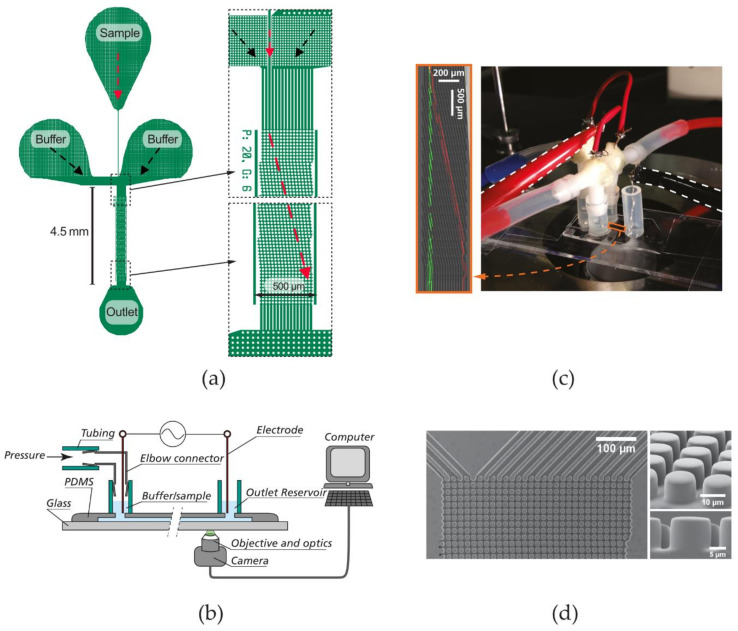
Devices and experimental setup. (**a**) Overview of device design. The red arrows schematically represent the trajectory of displacing particles. The actual trajectory angles of different devices are given in Table S2 of the Supplementary Information. Drawings are to scale. (**b**) Schematic of the experimental setup. (**c**) Image of the experimental setup, showing a DLD device on a microscope stage, with pressure and electrical connections. The electrical hooks are highlighted by the white dotted lines for visibility. The DLD array is enlarged and shown on the left, with color-coded trajectories of zigzagging (green) and displacing (red) particles. It has been compressed along its length by a factor of two to fit within the picture frame. (**d**) Scanning electron microscopic images of a typical DLD array (device #7, Table 1).

The DLD devices used throughout this paper were fabricated from polydimethylsiloxane (PDMS) using replica molding [88]. Detailed device fabrication steps can be found in Table S1 of the Supplementary Information. In each device (Figure 2a), only a narrow, single stream of sample is allowed to enter the DLD array and is buffered on two sides. Particles having different properties (size, charge) in the sample are separated as they travel along the array before entering a single outlet reservoir. At the observation windows at the beginning and at the end of the array, the lateral positions of the particles are analyzed.

A list of the devices used in this work is presented in Table 1, together with their featured parameters. More details about the designs of the devices can be found in Figure S1 and Table S2 of the Supplementary Information.

During an experiment, a device is positioned on the stage of an inverted microscope (Nikon Eclipse TE2000-U, Nikon Corporation, Tokyo, Japan) (Figure 2b,c). The pressure at the inlet reservoirs (1–100 mBar) is regulated by a pressure controller (MFCS-4C, Fluigent, Paris, France). The voltage applied along the device is generated by a function generator (33120A, Hewlett Packard, Palo Alto, CA, USA) and amplified using either a high-voltage amplifier (Bipolar Operational Power Supply/Amplifier BOP 1000M, Kepco, Flushing, NY, USA) or a high-frequency amplifier (WMA-300, Falco Systems, Amsterdam, The Netherlands). The voltage is measured using an oscilloscope (54603B 60MHz, Hewlett Packard, Palo Alto, CA, USA) with a 1×/10× probe (Kenwood PC-54, 600 V_PP_, Havant, UK). Note that care should be taken when working with high voltages. The voltage should be turned off before any changes are made to the setup. A pair of rubber gloves should be worn at all time during the course of the experiments.

To capture experimental image stacks, a monochrome Andor Neo sCMOS camera (Andor Technology, Belfast, Northern Ireland) is used. The image stacks are analyzed using FIJI (ImageJ 1.52f, National Institutes of Health, Bethesda, MD, USA) with MOSAIC particle tracking plugin [89]. The conductivities of the media are measured with a conductivity meter (B-771 LAQUAtwin, Horiba Instruments, Kyoto, Japan). The zeta potentials of the particles are measured with a Zetasizer NanoZS instrument (Malvern Instruments, Ltd., Worcestershire, UK).

### 2.2. Data Analysis

The sorting performance of a device can be assessed by comparing lateral positions of different types of particles at the beginning and at the end of the DLD array. In our experiments, the lateral positions are discretized by the gaps. To easily compare experiments across different devices, the lateral positions can be linearly converted into percentages, with 0% displacement being perfectly zigzagging and 100% displacement being perfectly displacing. The displacement of the particles can be compared between different experiments to study the effects of different parameters including voltage, frequency, pressure, and medium conductivity. More details of the data transforming processes can be found in Figure S4 of the Supplementary Information.

To quantify the particle displacement, we need to count the numbers of particles of interest exiting at each gap at the end (outlet) of the DLD arrays. This can be done manually, particle by particle, but this method is labor intensive and inapplicable for concentrated polystyrene nanospheres or nano liposomes. Instead, provided the particle concentration is not too high, which would lead to shadowing effects, the fluorescence intensity can be used to deduce the number of particles. More specifically, in the average image of all frames of an image stack, the integrated fluorescent intensity across each gap is proportional to the number of particles passing by that gap. We denote this quantity as *inferred particle count* in the plots throughout this paper. More details of the image processing can be found in Figure S2 of the Supplementary Information and a comparison between the method and manual count can be found in Figure S3.

### 2.3. Sample Preparation

Particle trajectories in the DLD devices were studied for a wide range of polystyrene beads and liposomes (see more details in Table S3 of the Supplementary Information).

*Polystyrene nano- and microspheres* (*D* = 160 nm–6.3 µm), referred to as beads for brevity, were suspended in media containing KCl at various molar concentrations and 0.1% *w*/*v* Pluronic^®^ F127 (Sigma-Aldrich Sweden AB, Stockholm, Sweden). Pluronic^®^ was found to markedly reduce non-specific sticking of the beads to PDMS. The volumetric concentrations of the beads were 0.02% *v*/*v* for 2 µm, 160 nm, 250 nm, and 490 nm beads, and 0.086% *v*/*v* for 4.3 µm and 6.3 µm beads.

*Liposomes* (*D* ~ 150–300 nm) were made by extrusion. Two lipids with different surface charge densities, the low-charge DOPC (1,2-dioleoyl-sn-glycero-3-phosphocholine) and the high-charge POPS (1-palmitoyl-2-oleoyl-sn-glycero-3-phospho-L-serine) were mixed to achieve liposomes with different zeta potentials. DOPC lipid was mixed with a lipid conjugated Rhodamine dye (18:1 Liss Rhod PE, 1,2-dioleoyl-sn-glycero-3-phosphoethanolamine-N-(lissamine rhodamine B sulfonyl) (ammonium salt)) in chloroform at concentrations of 10 mg/mL (DOPC) and 10 µg/mL (Rhodamine) in a glass vial. POPS lipid was used at a concentration of 1 mg/mL dissolved in chloroform. The lipids and the dye were purchased from Avanti Polar Lipids (Industrial Park Drive, Alabaster, AL, USA). The lipid solutions were gently blown with nitrogen until a dry, thin film formed at the bottom of the vial. A solution of KCl (2 mM) was added to the vial to obtain concentrations of DOPC, Rhodamine, and POPS of 1 mg/mL, 1 µg/mL, and 0.1 mg/mL, respectively, and the vial was vortexed vigorously to completely dissolve the lipid film into the solution. The solution was subsequently extruded 15 times back and forth through either 100 nm or 200 nm filters on an extruder platform (T&T Scientific Corporation, 7140 Regal Lane, Knoxville, TN 37918, USA). Finally, the extruded liposomes were characterized using the Zetasizer NanoZS instrument, for both size and zeta potential, before sorting experiments. All liposome samples are monodispersed, with polydispersity indices (PDI = (std/mean)^2^) below 0.24 (PDI_DOPC-100nm_ = 0.10, PDI_DOPC-200nm_ = 0.20, PDI_DOPC/POPS-100nm_ = 0.08, PDI_DOPC/POPS-200nm_ = 0.24), and no large clusters were observed in the size distributions from the DLS measurements.

## 3. Results and Discussion

To better understand the behavior of charged particles in an eDLD, we begin with the separation of polystyrene microspheres by their size and by their zeta potential. We then explore the capability of eDLD to effectively decrease the critical diameter to sort polystyrene nanospheres and nano-sized liposomes in micrometer-gap devices. In each case, the optimum conditions for separation regarding device geometry, applied pressure, electric field strength, and frequency are explored.

### 3.1. Important Sorting Parameters

We perform experiments on beads with surface carboxyl groups (4.3 µm and 6.3 µm), using device #5 with *D_C_* = 6.0 µm (for 4.3 µm beads) and device #6 with *D_C_* = 8.4 µm (for 6.3 µm beads) in order to determine contributions of some of the relevant sorting parameters.

**Figure 3:**
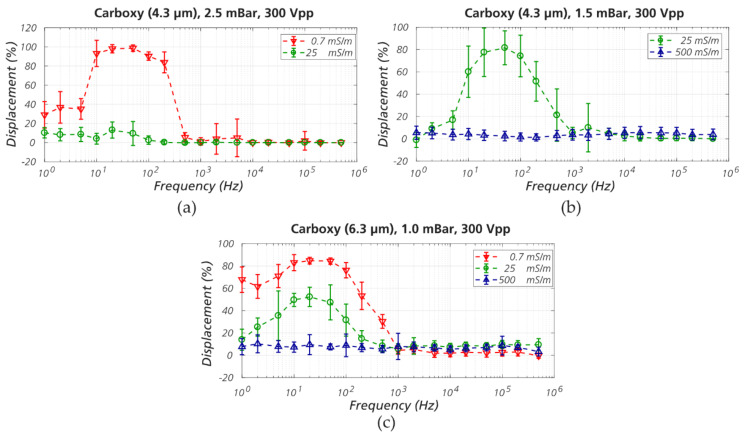
Effects of medium conductivity and voltage frequency on displacement of beads in eDLD. (**a**,**b**) The 4.3 µm beads in device #5 (*D_C_* = 6.0 µm) at 2.5 mBar and 1.5 mBar, respectively. The displacement is strongest at frequencies between 10 and 500 Hz, and at low electrical conductivity of the medium. (**c**) The 6.3 µm beads in device #6 (*D_C_* = 8.4 µm). The displacement is strongest at frequencies between 10 and 500 Hz, and at low conductivity.

*Effect of frequency and medium conductivity.* The applied voltage was kept constant (300 V_PP_), the applied pressure varied slightly (1.0, 1.5, or 2.5 mBar) over a distance of ≈ 7 mm between inlet and outlet reservoirs and the frequency scanned in the range 1 Hz-500 kHz. Results show (Figure 3) that the 4.3 µm and 6.3 µm beads are maximally displaced at frequencies below 1 kHz for all pressures and conductivities tested. Displacement was also observed to be greater at low medium conductivity (0.7 mS/m) and lower at high conductivity (500 mS/m). Since the zeta potential is known to decrease with increasing ionic strength [90] (see Equation (6) in Table S5 for details), this indicates that the enhanced displacement due to electrokinetics increases with |ζ|. For example, the absolute value of the particle zeta potential (|ζ|) of 4.3 µm beads at medium conductivity of 0.7 mS/m (milliQ water), 25 mS/m (1.7 mM KCl), and 500 mS/m (37 mM KCl) are 50 mV, 24 mV, and 1 mV, respectively. For 6.3 µm beads, they are 53 mV, 27 mV, and 1 mV, respectively (Table S3 of the Supplementary Information). This will be investigated further in Section 3.2.

**Figure 4:**
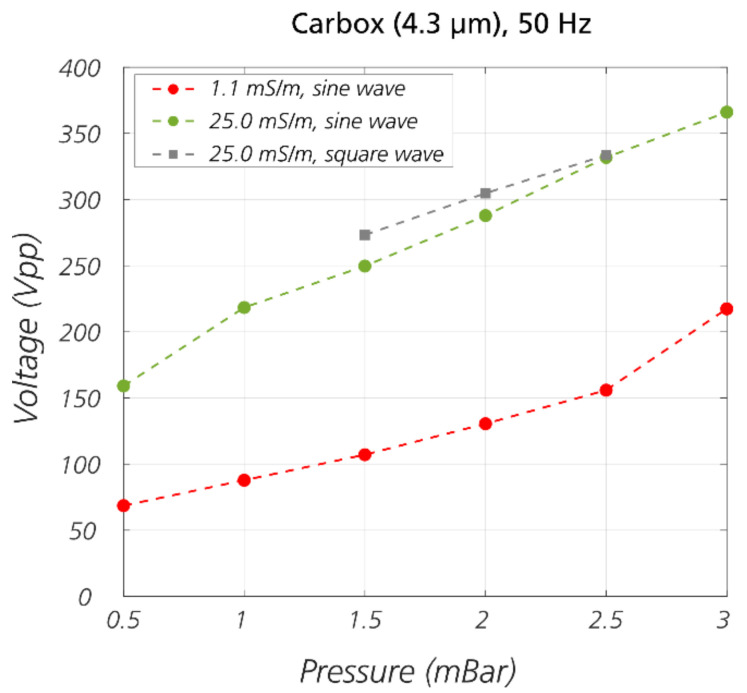
Effects of pressure and voltage on displacement of beads in eDLD. The data points are the voltage-pressure pairs that yield 50% displacement. More details about the data analysis can be found in Figure S4 of the Supplementary Information. The experiments were performed on 4.3 µm carboxylate beads in device #5 (*D_C_* = 6.0 µm) at 50 Hz, in media at conductivity of 1.1 mS/m or 25 mS/m. Note that the voltages of the square wave data points have been scaled up by a factor of $\sqrt{2}$, to compare with the sine wave data points.

*Effect of voltage and pressure.* Intuitively, the displacement increases when the applied voltage increases (the electrokinetic forces are stronger) or the applied pressure decreases (the particles travel slower, and the electrokinetic forces have more time to act). To confirm this, we kept the frequency constant at 50 Hz (which from Figure 3 gives the strongest displacement), and varied pressures from 0.5 mBar to 3 mBar in 0.5 mBar steps and applied voltages from 0 V_PP_ in 10–20 V_PP_ steps until the beads were completely displaced. The pressure/voltage combinations that yield 50% displacement, specifically, the threshold voltages required to displace beads to 50% at given pressures, are plotted in Figure 4.

It is apparent from the plot that at a higher applied pressure, a higher voltage is required in order to displace particles. What is more, the plot in Figure 4 confirms our previous observation that at lower medium conductivity, the effect is stronger and thus, lower voltage is required in order to displace particles at a given pressure. Since a square waveform gives a root mean square (RMS) voltage that is $\sqrt{2}$ greater than the RMS voltage of a sine wave, we performed experiments with a square waveform. Indeed, after applying a correction term of $\sqrt{2}$ the curves corresponding to the square and the sinewave overlap. It is important to note that a square wave consists of a sum of odd harmonics making the understanding of the frequency dependence less straightforward.

### 3.2. Sorting Polystyrene Microspheres by Zeta Potential

To study the effect of zeta potential, polystyrene microspheres of the same size (*D* = 2 µm) but different surface modifications were run in device #3 (*D_C_* = 2.8 µm). Although suspended in the same medium (25 mS/m KCl), the microspheres possess different surface charge density dependent on their types resulting in different zeta potentials (more details in Table S3 and Equation (6) of Table S5).

**Figure 5:**
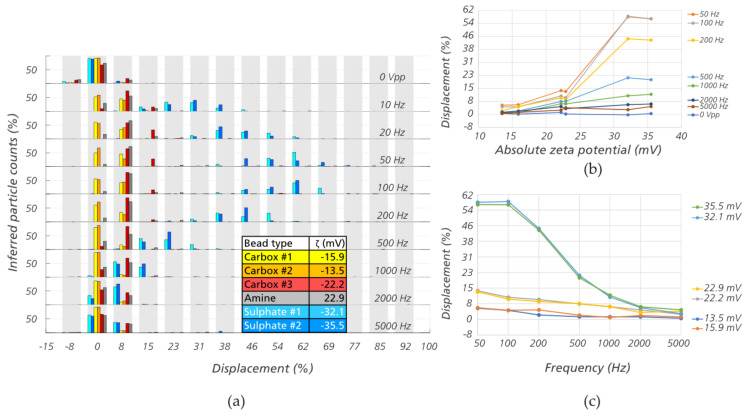
Displacement at outlet of 2 µm beads of different zeta potentials in device #3 (*D_C_* = 2.8 µm). (**a**) Displacement of six different bead types, at frequencies from 10 to 5000 Hz. (b) Displacement as a function of zeta potential. (**c**) Displacement as a function of frequency. In (**b**,**c**) the standard deviation of each data point corresponds approximately to 7% displacement (one gap). Note that there is small negative displacement in plot 5a at 0 V, which can be attributed to particle diffusion (see Section 5 and Table S4 of the Supplementary Information for the calculation of diffusion length).

The experiments were performed at constant pressure and voltage (*P* = 10 mBar and *V* = 300 V_PP_) applied over a distance of ≈ 7 mm between inlet and outlet reservoirs, while the frequency was varied from 10 Hz to 5 kHz. The displacement of different bead types at different frequencies are shown in Figure 5a. The distribution of displacements in Figure 5a can be fitted to normal distributions and the means are plotted against the zeta potentials (Figure 5b) and against the frequencies (Figure 5c). For illustration purposes, a supporting video showing the separation of the sulphate #2 (red) beads and carboxylate #2 (green) beads at 100 Hz can be found in the Supplementary Information (Video S1).

There is a clear correlation between the absolute value of the zeta potential and the bead displacement in Figure 5a,b. In the frequency range of 50–200 Hz, the sulphate beads, which have higher absolute values of zeta potential than that of the carboxylate beads and the amine beads, displace much more. This result demonstrates that same-sized particles with a difference in zeta potential on the order of 10 mV can be separated. Figure 5c confirms our observation in Section 3.1 that the effect is strong at low frequency (≤1 kHz).

The throughput of the devices was estimated to be 2 µL/hr with a Péclet number much larger than unity. Detailed calculations are presented in Table S4 of the Supplementary Information.

### 3.3. Sorting Polystyrene Nanospheres and Nano Liposomes

**Figure 6:**
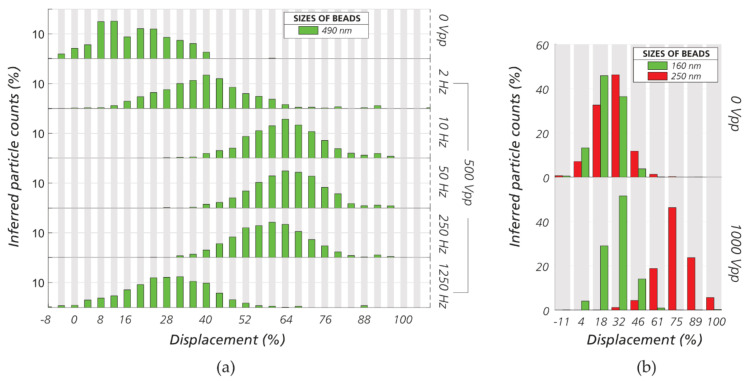
Displacement at outlet of nanospheres in micro-sized gap DLD devices. (**a**) Displacement of 490 nm beads in device #7 (*D_C_* = 1.24 µm) at 500 V_PP_. Medium: milliQ water + 0.1% Pluronic^®^ 127 (*σ* = 0.5 mS/m). (**b**) Sorting of 160 nm versus 250 nm beads in device #1 (*D_C_* = 0.66 µm) at 1000 V_PP_ at 1 kHz. Medium: milliQ water + 0.1% Pluronic^®^ 127 (*σ* = 0.5 mS/m).

We demonstrate sorting of nano particles in devices with micrometer sized gaps by displacing 490 nm beads in device #7 (*G* = 4 µm, *D_C_* = 1.24 µm) with 500 V_PP_ and 10 mBar applied over a distance of approximately ≈ 17 mm (Figure 6a). To decrease the size range further we used device #1 (*G* = 2 µm, *D_C_* = 0.66 µm) to separate beads of size 160 nm and 250 nm using 1000 V_PP_ and 7.5 mBar applied over a distance of ≈ 7 mm (Figure 6b). A supporting video can be found in the Supplementary Information (Video S2).

**Figure 7:**
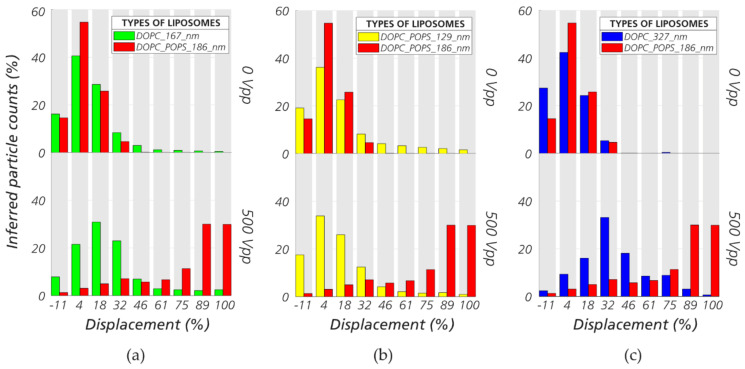
Sorting nano liposomes in eDLD. In general, the 1,2-dioleoyl-sn-glycero-3-phosphocholine (DOPC) liposomes possess lower charge than the DOPC+10%POPS liposomes. (**a**) Separation of liposomes of similar sizes but different surface properties ($\zeta_{\mathrm{DOPC},167\mathrm{nm}}=\;-10\;\mathrm{mV},{\;\zeta }_{\mathrm{DOPC}+10\%\mathrm{POPS},186\mathrm{nm}}=\;-67\;\mathrm{mV}$). The higher charged DOPC/1-palmitoyl-2-oleoyl-sn-glycero-3-phospho-L-serine (POPS) liposomes are more displaced. (**b**) Liposomes of different sizes but similar surface properties ($\zeta_{\mathrm{DOPC}+10\%\mathrm{POPS},129\mathrm{nm}}=\;-50\;\mathrm{mV},{\;\zeta }_{\mathrm{DOPC}+10\%\mathrm{POPS},186\mathrm{nm}}=\;-67\;\mathrm{mV}$). The larger liposomes are more displaced. (**c**) Large liposomes with lower zeta potential ($\zeta_{\mathrm{DOPC},327\mathrm{nm}}=\;-16\;\mathrm{mV}$) versus small liposomes with higher zeta potential ($\zeta_{\mathrm{DOPC}+10\%\mathrm{POPS},186\mathrm{nm}}=\;-67\;\mathrm{mV}$). In this case, the zeta potential is shown to be more important for the outcome of the sorting than the particle size. More information about size and zeta potential of liposomes can be found in Table S3 of the Supplementary Information. Device #1 (*D_C_* = 0.66 µm). Frequency: 1 kHz. Medium: KCl 2 mM (σ = 29 mS/m).

For nano liposome separation, we used device #1 (*G* = 2 µm, *D_C_* = 0.66 µm) to displace liposomes in the range 100–300 nm. We performed independent experiments on each of the four types of liposomes but in the same device and at the same voltage and pressure (500 V_PP_ and 10 mBar). The data are combined in the plots of Figure 7 for comparison. Our method allows us to separate by size (Figure 7b) or charge (Figure 7a,c) as the specific application requires. The full set of data relating to the sorting optimization of nanospheres and liposomes can be found in the Supplementary Information (Figure S5 and Figure S6, respectively). The sizes and zeta potentials of the nanospheres and nano liposomes can be found in Table S3 of the Supplementary Information.

### 3.4. The Role of Electrokinetic Driving Forces

By adding electrokinetics to DLD, its scope can be significantly broadened, which we demonstrate in the presented work. Additionally, we find the effect is only significant at low frequency (≤1 kHz) and increases with zeta potential of the particles. This suggests an association with the phenomena of electrophoresis (EP) and electroosmotic flow (EOF).

Electrophoresis and electroosmotic flow refer to the motion of charged particles and electrolyte solution, respectively, under an electric field. A charged particle suspended in an electrolyte solution will travel under the application of an electric field (particle EP). At the same time, the electrolyte solution contained in a channel with charged walls also moves when the field is on (EOF). In fact, particle EP stems from the EOF near the surface of the particle, so the two phenomena are related [41,43,91]. On a global scale, the total electrokinetic mobility of the particle is the algebraic sum of the electrophoretic mobility and the electroosmotic mobility. Under low-frequency AC fields, the particles can be seen oscillating along the direction of the field, as shown in Video S8 of the Supplementary Information.

**Figure 8:**
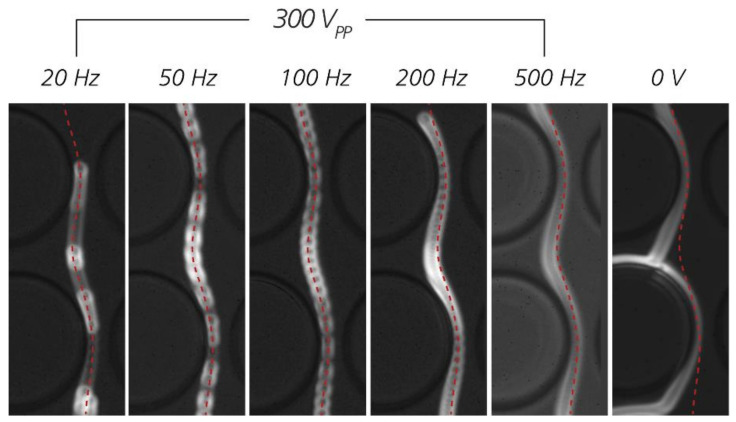
Comparison of trajectories of 2 µm carboxylate beads #1 at different frequencies. Identical red dotted lines based on trajectory at 100 Hz have been added to the images to guide the eye. Device #2 (*D_C_* = 2.3 µm) was used. The applied pressure was 30 mBar. The medium was KCl 22 mS/m with 2.5% *w/v* polyvinylpyrrolidone (PVP). Similar to Pluronic^®^ 127, PVP helps reduce non-specific adsorption of the beads to the PDMS walls of the device.

When a pressure is applied together with a low-frequency AC field, the particle can be seen traveling in the direction of the pressure gradient but at the same time oscillating at the frequency of the applied field. This can be seen in Video S4 of the Supplementary Information (pressure driven flow + AC 50 Hz), as compared to Video S3, where only a pressure was applied. The electrokinetic wall force depicted in Figure 1c can be observed in Video S5 (pressure driven flow + AC 10 Hz field) at time 0.34 s when the particle is “pushed” away from the pillar, leading to enhanced displacement. Note that the direction of this movement is perpendicular to the pillar and is different from the direction of the EP/EOF oscillation, which is tangential to the pillar (because the pillar is insulating, the electric field is tangential to the pillar). In Figure 8, trajectories of 2 µm carboxylate beads #1 at different frequencies are compared. It can be seen clearly that starting from 200 Hz, the beads displace less, which is consistent with the low-frequency operating range we have reported in Section 3.1 and Section 3.2.

At very low frequency (≤10 Hz), although the electrokinetic wall force might also be strong, the large oscillation magnitude of the particles may hinder the displacement. This can be observed in Video S6 (pressure driven flow + AC 2 Hz). During the negative phase of the oscillation, at around 1.00 sec, the particle is pulled backwards in the opposite direction to the pressure driven flow and may cross streamlines in the opposite direction to the displacement direction. This leads to particles moving in the zigzag mode while having a strong displacing tendency otherwise. This explains the lower displacement of particles at very low frequencies in the plots of Figure 3.

The prospects of various electrokinetic mechanisms influencing sorting in DLD devices have been brought up for discussion in the literature [80,81]. Due to viscous damping, EOF and EP is not expected to be of importance at higher frequencies. Calero et al. [80] identified 500 Hz as a reasonable threshold frequency, above which EOF and EP would not be important.

It is at this point relevant to mention that there are two types of EOF: *Linear* and *non-linear EOF* [92]. In linear EOF, the external field is much smaller than the field in the electric double layer (EDL) (which can be estimated by the zeta potential and the Debye length, and is on the order of 10^6^ V/m). The external field therefore does not affect the distribution of charge in the EDL. With this condition, the velocity of the fluid or a particle can be calculated by Smoluchowski’s equation:

(2) $$v=\frac{\zeta \varepsilon }{\eta }E^{\;}$$

where ζ is the zeta potential of the wall or particle, ε is the permittivity of the fluid, η is the viscosity of the fluid, and ***E*** is the external field. If ζ>0, v is parallel with E for EP and anti-parallel with E for EOF.

On the contrary, if the external field gives rise to or modifies the charge distribution in the EDL, ζ in Equation (2) is no longer independent of ***E*** and the velocity no longer varies linearly with the external field. There are several embodiments of this non-linear electrokinetics reported in the literature. For instance, when the external field polarizes conductive walls (metal electrodes), it exerts at the same time an electric force on the newly induced charge and causes circulation of fluid just above the electrodes. This phenomenon is called *AC-electroosmosis* [93,94,95] and the time-averaged electroosmotic velocity is proportional to the square of the applied voltage [96]:

(3) $$\langle v\rangle \propto \frac{\varepsilon }{\eta }f\left(\omega ,\varepsilon ,\sigma ,\kappa ,z\right)V^{2}$$

where *f* is a function of angular frequency *ω*, medium permittivity *ε,* medium conductivity *σ,* inverse of Debye length *κ,* and distance *z* from electrodes.

Similarly, for the case of conducting particles (or in 2D, a rod), one can observe vortices near the particle surface. This phenomenon is called *induced-charge electroosmosis/electrophoresis (ICEO/ICEP)* [97,98] and the typical flow speeds are also proportional to the square of the applied field:

(4) $$U_{0}\propto \frac{\varepsilon }{\eta }aE^{2}$$

**Figure 9:**
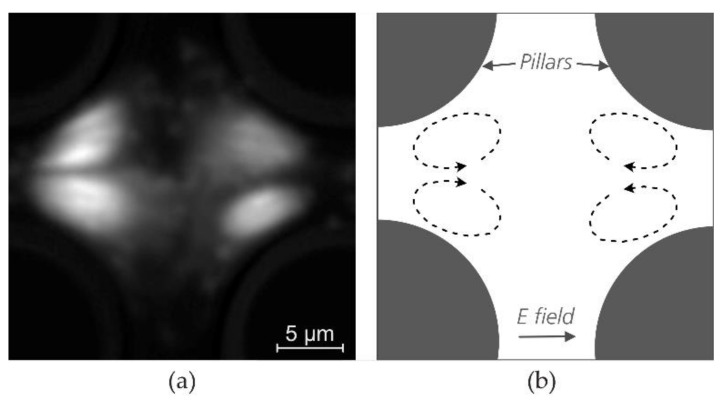
Vortices of 1.1 µm polystyrene beads in milliQ water at 500 V_PP_ at 100 Hz, in device #4 (*G* = 8 µm, distance between electrodes ≈ 7 mm). (**a**) Actual image, (**b**) Diagram showing the direction of the vortices). The original image stack (*557* frames @25fps) has the field of view of 8 × 7 identical unit cells (the image shows one unit cell). To simultaneously observe the dynamics of many particles, we used MATLAB 2017a (Mathworks, Natick, MA, USA) to superimpose all these unit cells into one unit cell. The resulted image stack was then averaged to produce the image shown in the figure. A video with particle tracking is available in the Supplementary Information (Video S7).

Non-linear electrokinetics for dielectrics, which is more relevant to our work, has also been studied [99,100]. Notably, Wang et al. [100] observed vortices of 1 µm polystyrene particles near a narrow constriction when a 1 kHz AC field was applied, which they attributed to ICEO and electrothermal flow (the former dominated in low conductivity medium). We also observe vortices of 1.1 µm polystyrene particles suspended in milliQ water in a pillar array under 100 Hz AC field (Figure 9 and Supplementary Information Video S7) when no pressure driven flow is present. Similar vortices have also been reported by Calero et al. [81] in their type of eDLD with microelectrodes on the sides of the device.

**Figure 10:**
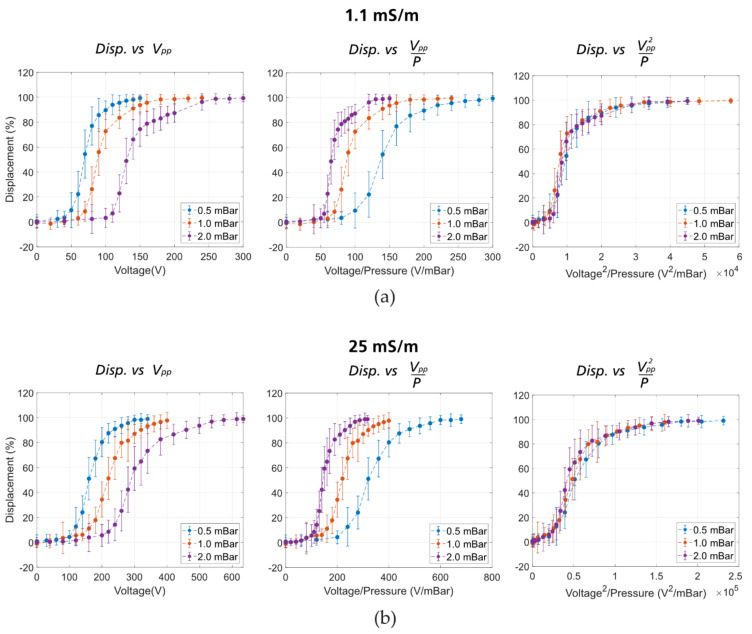
Displacement of 4.3 µm beads as a function of voltage, voltage/pressure, or voltage^2^/pressure. (**a**) At medium conductivity of 1.1 mS/m. (**b**) At medium conductivity of 25 mS/m.

In our eDLD experiments, since the enhanced displacement effect (from now on called “the effect”) decreases with increasing medium conductivity at any given applied voltage, electrothermal flow can be excluded. Since the effect is significant at low frequency and increases with the zeta potential, it may have some dependence on EP and EOF (either linear or non-linear). To investigate this, we look into the displacement of 4.3 µm beads at different applied pressures and voltages, and at two different conductivities (Figure 10). For each conductivity, the plot on the left shows the displacement as a function of the voltage. These plots confirm that at higher pressure, a higher voltage is required to achieve the same displacement as in the low-pressure case. This is because the particles travel faster, and the electrokinetic forces have less time to act at higher pressure.

To compare different experiments, the pressure can be normalized (the middle plot for each conductivity in Figure 10). In these plots, if the effect is linearly dependent on the voltage, the data at different pressures should collapse into one curve (see Section 6 in the Supplementary Information for a simplified mathematical model). It is shown not to be the case. However, when we plot the displacement as a function of voltage squared over pressure (the plot on the right for each conductivity of Figure 10), the data collapse to one curve within the uncertainty of our experiments. This suggests association with non-linear electrokinetics, which varies with the square of the applied voltage, as shown in Equations (3) and (4).

**Figure 11:**
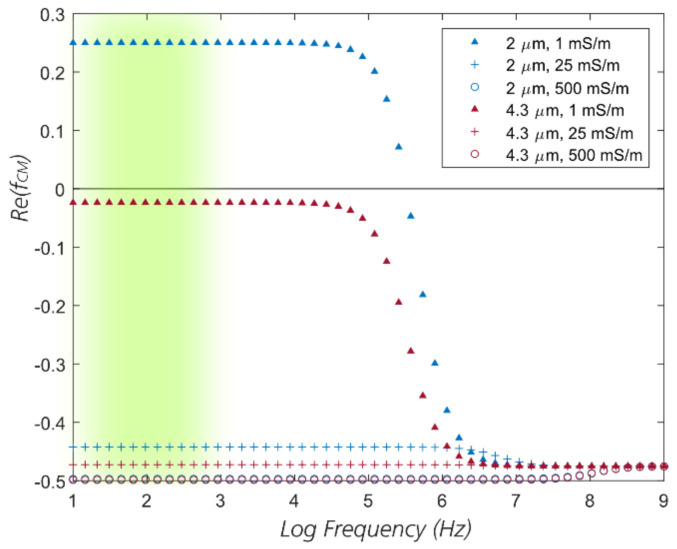
The real part of the Clausius–Mossotti factor for 2 µm and 4.3 µm beads at different conductivities used in the experiments, assuming *K* = 1 nS for all beads. The blurred green region highlights the range of frequencies where we observe the enhanced displacement effect.

It is necessary to discuss dielectrophoresis (DEP) as a possible mechanism since the DEP force is also proportional to *V*^2^. The DEP force on a spherical particle of radius *r* in a non-uniform electric field ***E*** is given by:

(5) $$F_{DEP}=\;2\pi \varepsilon_{m}r^{3}Re\left(\frac{{\tilde{\varepsilon }}_{p}-{\tilde{\varepsilon }}_{m}}{{\tilde{\varepsilon }}_{p}+2{\tilde{\varepsilon }}_{m}}\right)\nabla {\left|E_{rms}\right|}^{2}$$

where $E_{rms}$ is the root mean square value of the field, ${\tilde{\varepsilon }}_{p}$, ${\tilde{\varepsilon }}_{m}$ are the complex permittivity of the particle (*p*) and the suspending medium (*m*) and are defined as: $\tilde{\varepsilon }=\varepsilon -i\sigma /\omega .$ Here ε is the permittivity, *σ* is the conductivity, and *ω* is the angular frequency of the electric field. The term in the brackets represents the relative contrast between the permittivities of the particle and the medium and is called the Clausius–Mossotti factor: $f_{CM}=\frac{{\tilde{\varepsilon }}_{p}-{\tilde{\varepsilon }}_{m}}{{\tilde{\varepsilon }}_{p}+2{\tilde{\varepsilon }}_{m}}$. For polystyrene beads, the bulk conductivity $\sigma_{b}$ is negligibly small and their conductivity comes from the surface conductivity $\sigma_{s}$ [101]:

(6) $$\sigma_{p}\approx \sigma_{s}=\frac{2K}{r}$$

where K is the surface conductance of the beads. For non-conducting polystyrene particles, typically K∼1nS [102,103]. According to Equation (5), the polarity of the DEP force is dependent on the real part of the Clausius–Mossotti factor. When $Re\left(f_{CM}\right)>0$, the particles travel to the regions of high field strength (positive or pDEP), conversely when $Re\left(f_{CM}\right)<0$, the particles travel to the regions of low field strength (negative or nDEP). In eDLD with the electric field parallel to the flow field, as has been pointed out by Beech et al. [77], the nDEP force “pushes” the particles away from the pillar out of the first stream and, if sufficiently strong, into the subsequent stream (i.e., in same direction as $F_{EW}$ in Figure 1c). Conversely, pDEP “pulls” the particles towards the pillar, which intuitively reduces the effect. To assess the contribution of DEP, the real parts of the Clausius–Mossotti factors for 2 µm and 4.3 µm polystyrene beads at different conductivities used in this paper are plotted in Figure 11.

Figure 11 shows that at high frequencies (≥1 MHz), the CM factor approaches a value close to −0.5 asymptotically, giving rise to nDEP. At lower frequencies it is positive (pDEP) for small particles in low conductivity medium (here only for 2 µm beads in milliQ water) and nDEP for large particles or high medium conductivities relative to the particle conductivity. In our experimental data presented in Section 3.1 and Section 3.2, we observe a significantly enhanced displacement effect only at frequencies between 10 Hz and 1 kHz (the green region in Figure 11) whereas the DEP force, whether it is pDEP or nDEP, is constant in a much broader frequency range, from 10 Hz to 100 kHz. This indicates that DEP is not the main contribution to the effect and suggests that non-linear EOF or EP probably plays a more important role. It should be noted that nDEP can be utilized to enhance particle displacement but required applied voltages might be higher than those used here.

Finally, we consider whether or not it is possible to improve future devices based on the findings of the present study. The question can only be answered in relation to the specific requirements of the application, but we can give some general guidelines. Larger devices can provide higher throughput. However, higher applied voltages are required in order to maintain the necessary field strengths inside pillar arrays, which constitutes a practical upper limit on device size. It is possible to increase the field gradients inside a pillar array for a given applied voltage, which can improve device performance, but these improvements also come at a cost. For example, simulations (see Figure S8c of the Supplementary Information) show that changing the gap (*G)* and the period (*N*) such that the critical size remains the same does not have a significant effect on the field strength at one critical radius distance from the posts, and we conclude that these parameters alone cannot be used to greatly improve the voltage response in the device. Changing the diameters (*P*) of the posts however has a larger impact on the local field strength for a given applied voltage (see Figure S8d of the Supplementary Information). This can be understood by considering the ratio *P/G,* which indicates the amount by which the electric field is constricted between the posts. Although the field strength at one critical radius distance from the posts increases 35% when *P/G* goes from 0.28 to 3.3 with *Dc* kept constant, which is significant, the device would also need to be considerably longer, 3.4 times longer in this case, which entails a large increase in the required applied voltage. Since large *N* also leads to longer devices for a given critical size, in general, small *N* and large *P/G* lead to higher fields for a given applied voltage. Other changes to both the geometry of the post array and to the shapes of the posts are expected to change the distribution of the electric field in the post array and could also be used to optimize device performance for specific applications, but that is outside the scope of the present work and will be explored in future studies.

## 4. Conclusions

We have presented an integration of DLD and electrokinetics to sort particles based not only on size, but also on zeta potential which in turn depends on the surface charge. The integration has been done in the simplest way possible, with electrodes located in inlet and outlet reservoirs. Without having to fabricate microelectrodes or scale down the devices, we were able to sort nanoparticles in devices having micrometer-sized gaps, in a way that is sensitive to size and zeta potential. Our approach is applicable to all particle sizes from the micro- down to the nanoscale and is not bound by requirements for very low salt concentrations for the running medium.

We posit that non-linear electroosmotic flow and electrophoresis play an important role in the mechanism of zeta-potential-based or charge-based sorting using eDLD. However, further studies are necessary to fully understand how these mechanisms interact and are responsible for the separation. Nevertheless, we have demonstrated that by optimizing running parameters such as ionic strength, applied voltage, frequency, and pressure, we can adapt the approach to a wide range of relevant particle sizes and zeta potentials.

Our results prove that eDLD can be used to sort nano particles such as liposomes with strong biological relevance, for example in medicine and pharmacology, most notably for drug delivery. This opens up for sorting extracellular vesicles (EVs), which are similar in structure to liposomes. EVs have great potential in diagnostics and therapeutics but current EV manufacturing and sorting methods are inadequate in terms of purity, reproducibility, yield, time, and cost [84,85]. From a fabrication perspective our eDLD devices are simple, disposable, and can be realized in cheap materials using standard techniques. Ongoing work is focused on using what we have learned to developing devices that will be capable of sorting EVs based on size and surface charge, at throughputs relevant for further bioanalysis.

## Figures and Tables

**Table 1:** Parameters of DLD devices used in this work. Critical diameters, *D_C_*, are nominal values based on the geometry of the pillar array (Equation (1)).

Device Name	Gap (µm)	*N*	*D_C_* (µm)
Device #1	2	20	0.66
Device #2	5	10	2.32
Device #3	6	10	2.78
Device #4	8	10	3.71
Device #5	13	10	6.03
Device #6	13	5	8.41
Device #7	4	23	1.24

## Supplementary Materials

The following are available online at https://www.mdpi.com/2072-666X/11/11/1014/s1: **Figure S1**: Overview of a typical device, **Figure S2**: Image processing steps used to estimate relative particle counts based on fluorescence intensity, **Figure S3**: Relative amounts of 2 µm beads as measured by manual counting and by integrated fluorescent intensity, **Figure S4**: Summary of data transforming steps in order to investigate the effects of different running parameters on particle displacement, **Figure S5**: Optimization of nanosphere sorting, **Figure S6**: Optimization of liposome sorting, with voltage as the changing parameter, **Figure S7**: Simple sketch demonstrating the displacement caused by electrokinetic force, **Figure S8**: Electric field simulation of eDLD, **Table S1**: Device fabrication steps, **Table S2**: Parameters of DLD devices used in this work, **Table S3**: Specifications of the particles used in this work, **Table S4**: Throughput and Péclet numbers of the experiments reported in the main text, **Table S5**: Important equations of electrokinetics, **Video S1**: Sorting—Sulphate2 (red)—Carbox2 (green)—100 Hz. *Real-time, color-coded video showing the separation of the sulphate #2 (red) and carboxylate #2 (green) beads (both ≈ 2µm) in device #3 (D_C_ = 2.8 µm) when the electric field was turned on at the middle of the video. The result is plotted in Figure 5 (main text).* **Video S2**: Sorting—Red 250 nm—Green 160 nm—0 V_PP_ then 1000 V_PP_—1000 Hz. *Sorting of 250 nm and 160 nm beads at an applied voltage of 1000 V_PP_ at 1 kHz. The separation is possible only after the voltage was turned on. The playback is fast-forwarded twice.* **Video S3**: Trajectory—4um3 particle under pressure driven flow. *Trajectories of of a 4.3 µm bead in device #5 (D_C_ = 6.0 µm), under a pressure of 1.5 mBar, in KCl 25 mS/m with 0.1% w/v Pluronic^®^ 127. The pressure driven flow is from left to right. The playback is slowed down 10x.* **Video S4–S6**: Similar to Video S3 but with voltage of 300 V_PP_/square wave at frequencies of 50 Hz, 10 Hz, and 2 Hz, respectively. *The playback is slowed down 40x, 40x, and 10x, respectively. The pressure driven flow is from left to right. The oscillatory motion of beads can be seen clearly in Video S4 (50 Hz) and Video S5 (10 Hz). The enhanced displacement can be seen clearly in Video S5 (10 Hz) at 0.34 s. The setback from displacing to zigzag at very low frequency can be seen in Video S6 (2 Hz) at 1.00 sec. Note that we used square wave in these slow-motion videos, instead of the sine wave as in most of the data in the main text, to give the eyes an easier detection of the phase of the oscillation.* **Video S7**: Trajectory—1um1 particles under 100 Hz AC signal (no pressure driven flow). *Vortexes of 1.1 µm beads in milliQ water at 500 V_PP_ at 100 Hz, in device #4 (G = 8 µm). The image is combined from 557 frames @25fps of 8 × 4 different unit cells (i.e., the full field of view is 32 times as wide as the video dimensions).* **Video S8**: Oscillation—2um particles—100Hz (red) 500Hz (green) 5000Hz (magenta)—(superimposed) .mp4 (no pressure driven flow). *Superimposed, color-coded video showing the oscillatory motion of the same type of bead at different frequencies, in the gap between two pillars, when an AC voltage was applied from left to right (no pressure applied). The video was combined from three videos at three different frequencies. The video has been slowed down by 50 times.*

## References

[1] Huang L.R., Cox E.C., Austin R.H., Sturm J.C. (2004). Continuous particle separation through deterministic lateral displacement. Science.

[2] Siyang Z., Yung R., Yu-Chong T., Kasdan H. Deterministic lateral displacement MEMS device for continuous blood cell separation. Proceedings of the 18th IEEE International Conference on Micro Electro Mechanical Systems.

[3] Davis J.A., Inglis D.W., Morton K.J., Lawrence D.A., Huang L.R., Chou S.Y., Sturm J.C., Austin R.H. (2006). Deterministic hydrodynamics: Taking blood apart. Proc. Natl. Acad. Sci. USA.

[4] Li N., Kamei D.T., Ho C.M. On-chip continuous blood cell subtype separation by deterministic lateral displacement. Proceedings of the 2007 2nd IEEE International Conference on Nano/Micro Engineered and Molecular Systems.

[5] Inglis D.W., Lord M., Nordon R.E. (2011). Scaling deterministic lateral displacement arrays for high throughput and dilution-free enrichment of leukocytes. J. Micromechanics Microengineering.

[6] Holmes D., Whyte G., Bailey J., Vergara-Irigaray N., Ekpenyong A., Guck J., Duke T. (2014). Separation of blood cells with differing deformability using deterministic lateral displacement. Interface Focus.

[7] Loutherback K., D’Silva J., Liu L., Wu A., Austin R.H., Sturm J.C. (2012). Deterministic separation of cancer cells from blood at 10 mL/min. AIP Adv..

[8] Liu Z., Huang F., Du J., Shu W., Feng H., Xu X., Chen Y. (2013). Rapid isolation of cancer cells using microfluidic deterministic lateral displacement structure. Biomicrofluidics.

[9] Karabacak N.M., Spuhler P.S., Fachin F., Lim E.J., Pai V., Ozkumur E., Martel J.M., Kojic N., Smith K., Chen P.I. (2014). Microfluidic, marker-free isolation of circulating tumor cells from blood samples. Nat. Protoc..

[10] Okano H., Konishi T., Suzuki T., Suzuki T., Ariyasu S., Aoki S., Abe R., Hayase M. (2015). Enrichment of circulating tumor cells in tumor-bearing mouse blood by a deterministic lateral displacement microfluidic device. Biomed. Microdevices.

[11] Au S.H., Edd J., Stoddard A.E., Wong K.H.K., Fachin F., Maheswaran S., Haber D.A., Stott S.L., Kapur R., Toner M. (2017). Microfluidic isolation of circulating tumor cell clusters by size and asymmetry. Sci. Rep..

[12] Liu Z., Chen R., Li Y., Liu J., Wang P., Xia X., Qin L. (2018). Integrated microfluidic chip for efficient isolation and deformability analysis of circulating tumor cells. Adv. Biosyst..

[13] Holm S.H., Beech J.P., Barrett M.P., Tegenfeldt J.O. (2011). Separation of parasites from human blood using deterministic lateral displacement. Lab Chip.

[14] Holm S.H., Beech J.P., Barrett M.P., Tegenfeldt J.O. (2016). Simplifying microfluidic separation devices towards field-detection of blood parasites. Anal. Methods.

[15] Huang R., Barber T.A., Schmidt M.A., Tompkins R.G., Toner M., Bianchi D.W., Kapur R., Flejter W.L. (2008). A microfluidics approach for the isolation of nucleated red blood cells (NRBCs) from the peripheral blood of pregnant women. Prenat. Diagn..

[16] Laki A.J., Botzheim L., Ivan K., Szabo T., Tamasi V., Buzas E., Civera P. Microvesicle fractionation using deterministic lateral displacement effect. Proceedings of the 9th IEEE International Conference on Nano/Micro Engineered and Molecular Systems (NEMS).

[17] Laki A.J., Botzheim L., Ivan K., Tamasi V., Civera P. (2015). Separation of microvesicles from serological samples using deterministic lateral displacement effect. Bionanoscience.

[18] D’Silva J., Austin R.H., Sturm J.C. (2015). Inhibition of clot formation in deterministic lateral displacement arrays for processing large volumes of blood for rare cell capture. Lab Chip.

[19] Green J.V., Radisic M., Murthy S.K. (2009). Deterministic lateral displacement as a means to enrich large cells for tissue engineering. Anal. Chem..

[20] Zhang B., Green J.V., Murthy S.K., Radisic M. (2012). Label-free enrichment of functional cardiomyocytes using microfluidic deterministic lateral flow displacement. PLoS ONE.

[21] Liu Z., Lee Y., Jang J., Li Y., Han X., Yokoi K., Ferrari M., Zhou L., Qin L. (2015). Microfluidic cytometric analysis of cancer cell transportability and invasiveness. Sci. Rep..

[22] Tottori N., Nisisako T., Park J., Yanagida Y., Hatsuzawa T. (2016). Separation of viable and nonviable mammalian cells using a deterministic lateral displacement microfluidic device. Biomicrofluidics.

[23] Xavier M., Holm S.H., Beech J.P., Spencer D., Tegenfeldt J.O., Oreffo R.O.C., Morgan H. (2019). Label-free enrichment of primary human skeletal progenitor cells using deterministic lateral displacement. Lab Chip.

[24] Inglis D.W., Herman N., Vesey G. (2010). Highly accurate deterministic lateral displacement device and its application to purification of fungal spores. Biomicrofluidics.

[25] Joensson H.N., Uhlen M., Svahn H.A. (2011). Droplet size based separation by deterministic lateral displacement-separating droplets by cell—Induced shrinking. Lab Chip.

[26] Tottori N., Hatsuzawa T., Nisisako T. (2017). Separation of main and satellite droplets in a deterministic lateral displacement microfluidic device. RSC Adv..

[27] Beech J.P., Ho B.D., Garriss G., Oliveira V., Henriques-Normark B., Tegenfeldt J.O. (2018). Separation of pathogenic bacteria by chain length. Anal. Chim. Acta.

[28] Wunsch B.H., Kim S.C., Gifford S.M., Astier Y., Wang C., Bruce R.L., Patel J.V., Duch E.A., Dawes S., Stolovitzky G. (2019). Gel-on-a-chip: Continuous, velocity-dependent DNA separation using nanoscale lateral displacement. Lab A Chip.

[29] Wunsch B.H., Smith J.T., Gifford S.M., Wang C., Brink M., Bruce R.L., Austin R.H., Stolovitzky G., Astier Y. (2016). Nanoscale lateral displacement arrays for the separation of exosomes and colloids down to 20 nm. Nat. Nanotechnol..

[30] McGrath J., Jimenez M., Bridle H. (2014). Deterministic lateral displacement for particle separation: A review. Lab Chip.

[31] Salafi T., Zhang Y., Zhang Y. (2019). A review on deterministic lateral displacement for particle separation and detection. Nano Micro Lett..

[32] Zeming K.K., Thakor N.V., Zhang Y., Chen C.H. (2016). Real-time modulated nanoparticle separation with an ultra-large dynamic range. Lab Chip.

[33] Mutlu B.R., Smith K.C., Edd J.F., Nadar P., Dlamini M., Kapur R., Toner M. (2017). Non-equilibrium inertial separation array for high-throughput, large-volume blood fractionation. Sci. Rep..

[34] Frechette J., Drazer G. (2009). Directional locking and deterministic separation in periodic arrays. J. Fluid Mech..

[35] Davis J.A. (2008). Microfluidic Separation of Blood Components through Deterministic Lateral Displacement. Ph.D. Thesis.

[36] Beech J.P., Holm S.H., Adolfsson K., Tegenfeldt J.O. (2012). Sorting cells by size, shape and deformability. Lab Chip.

[37] Jiang M.L., Budzan K., Drazer G. (2015). Fractionation by shape in deterministic lateral displacement microfluidic devices. Microfluid. Nanofluidics.

[38] Henry E., Holm S.H., Zhang Z., Beech J.P., Tegenfeldt J.O., Fedosov D.A., Gompper G. (2016). Sorting cells by their dynamical properties. Sci. Rep..

[39] Calero V., Garcia-Sanchez P., Ramos A., Morgan H. (2019). Combining DC and AC electric fields with deterministic lateral displacement for micro- and nano-particle separation. Biomicrofluidics.

[40] Henry D. (1931). The cataphoresis of suspended particles. Part I.—The equation of cataphoresis. Proc. R. Soc. Lond. Ser. A Contain. Pap. A Math. Phys. Character.

[41] Wiersema P.H., Loeb A.L., Overbeek J.T.G. (1966). Calculation of the electrophoretic mobility of a spherical colloid particle. J. Colloid Interface Sci..

[42] Morrison Jr F.A. (1970). Electrophoresis of a particle of arbitrary shape. J. Colloid Interface Sci..

[43] Viovy J.L. (2000). Electrophoresis of DNA and other polyelectrolytes: Physical mechanisms. Rev. Mod. Phys..

[44] Pohl H.A. (1978). Dielectrophoresis: The Behavior of Neutral Matter in Nonuniform Electric Fields (Cambridge Monographs on Physics).

[45] Pethig R. (2010). Review article-dielectrophoresis: Status of the theory, technology, and applications. Biomicrofluidics.

[46] Pethig R.R. (2017). Dielectrophoresis: Theory, Methodology and Biological Applications.

[47] Pohl H.A., Hawk I. (1966). Separation of living and dead cells by dielectrophoresis. Science.

[48] Markx G.H., Talary M.S., Pethig R. (1994). Separation of viable and non-viable yeast using dielectrophoresis. J. Biotechnol..

[49] Markx G.H., Pethig R. (1995). Dielectrophoretic separation of cells: Continuous separation. Biotechnol. Bioeng.

[50] Church C., Zhu J., Wang G., Tzeng T.R., Xuan X. (2009). Electrokinetic focusing and filtration of cells in a serpentine microchannel. Biomicrofluidics.

[51] Kang Y., Li D., Kalams S.A., Eid J.E. (2008). DC-Dielectrophoretic separation of biological cells by size. Biomed. Microdevices.

[52] Gallo-Villanueva R.C., Jesus-Perez N.M., Martinez-Lopez J.I., Pacheco A., Lapizco-Encinas B.H. (2011). Assessment of microalgae viability employing insulator-based dielectrophoresis. Microfluid. Nanofluidics.

[53] Lapizco-Encinas B.H., Simmons B.A., Cummings E.B., Fintschenko Y. (2004). Dielectrophoretic concentration and separation of live and dead bacteria in an array of insulators. Anal. Chem..

[54] Lapizco-Encinas B.H., Simmons B.A., Cummings E.B., Fintschenko Y. (2004). Insulator-based dielectrophoresis for the selective concentration and separation of live bacteria in water. Electrophoresis.

[55] Braff W.A., Willner D., Hugenholtz P., Rabaey K., Buie C.R. (2013). Dielectrophoresis-based discrimination of bacteria at the strain level based on their surface properties. PLoS ONE.

[56] Morgan H., Hughes M.P., Green N.G. (1999). Separation of submicron bioparticles by dielectrophoresis. Biophys. J..

[57] Chou C.F., Tegenfeldt J.O., Bakajin O., Chan S.S., Cox E.C., Darnton N., Duke T., Austin R.H. (2002). Electrodeless dielectrophoresis of single- and double-stranded DNA. Biophys. J..

[58] Jones P.V., Salmon G.L., Ros A. (2017). Continuous separation of dna molecules by size using insulator-based dielectrophoresis. Anal. Chem..

[59] Liao K.T., Tsegaye M., Chaurey V., Chou C.F., Swami N.S. (2012). Nano-constriction device for rapid protein preconcentration in physiological media through a balance of electrokinetic forces. Electrophoresis.

[60] Abdallah B.G., Chao T.C., Kupitz C., Fromme P., Ros A. (2013). Dielectrophoretic sorting of membrane protein nanocrystals. ACS Nano.

[61] Lapizco-Encinas B.H. (2019). On the recent developments of insulator-based dielectrophoresis: A review. Electrophoresis.

[62] Price J.A., Burt J.P., Pethig R. (1988). Applications of a new optical technique for measuring the dielectrophoretic behaviour of micro-organisms. Biochim. Biophys. Acta.

[63] Pethig R., Huang Y., Wang X.B., Burt J.P.H. (1992). Positive and negative dielectrophoretic collection of colloidal particles using interdigitated castellated microelectrodes. J. Phys. D Appl. Phys..

[64] Huang Y., Pethig R. (1991). Electrode design for negative dielectrophoresis. Meas. Sci. Technol..

[65] Hoettges K.F., Hughes M.P., Cotton A., Hopkins N.A., McDonnell M.B. (2003). Optimizing particle collection for enhanced surface-based biosensors. IEEE Eng. Med. Biol. Mag..

[66] Hoettges K.F., Hubner Y., Broche L.M., Ogin S.L., Kass G.E., Hughes M.P. (2008). Dielectrophoresis-activated multiwell plate for label-free high-throughput drug assessment. Anal. Chem..

[67] Masuda S., Washizu M., Nanba T. (1989). Novel method of cell fusion in field constriction area in fluid integration circuit. IEEE Trans. Ind. Appl..

[68] Pysher M.D., Hayes M.A. (2007). Electrophoretic and dielectrophoretic field gradient technique for separating bioparticles. Anal. Chem..

[69] Braff W.A., Pignier A., Buie C.R. (2012). High sensitivity three-dimensional insulator-based dielectrophoresis. Lab Chip.

[70] Barrett L.M., Skulan A.J., Singh A.K., Cummings E.B., Fiechtner G.J. (2005). Dielectrophoretic manipulation of particles and cells using insulating ridges in faceted prism microchannels. Anal. Chem..

[71] Hawkins B.G., Smith A.E., Syed Y.A., Kirby B.J. (2007). Continuous-flow particle separation by 3D insulative dielectrophoresis using coherently shaped, dc-biased, ac electric fields. Anal. Chem..

[72] Zhu J., Xuan X. (2009). Particle electrophoresis and dielectrophoresis in curved microchannels. J. Colloid Interface Sci..

[73] Cummings E.B., Singh A.K. Dielectrophoretic trapping without embedded electrodes. Proceedings of the SPIE: Conference on Microfluidic Devices and Systems III.

[74] Cummings E.B., Singh A.K. (2003). Dielectrophoresis in microchips containing arrays of insulating posts: Theoretical and experimental results. Anal. Chem..

[75] Camacho-Alanis F., Gan L., Ros A. (2012). Transitioning streaming to trapping in DC insulator-based dielectrophoresis for biomolecules. Sens. Actuators B Chem..

[76] Hanasoge S., Devendra R., Diez F.J., Drazer G. (2015). Electrokinetically driven deterministic lateral displacement for particle separation in microfluidic devices. Microfluid. Nanofluidics.

[77] Beech J.P., Jonsson P., Tegenfeldt J.O. (2009). Tipping the balance of deterministic lateral displacement devices using dielectrophoresis. Lab Chip.

[78] Tran T.S.H., Ho B.D., Beech J.P., Tegenfeldt J.O. (2017). Open channel deterministic lateral displacement for particle and cell sorting. Lab Chip.

[79] Beech J.P., Keim K., Ho B.D., Guiducci C., Tegenfeldt J.O. (2019). Active posts in deterministic lateral displacement devices. Adv. Mater. Technol..

[80] Calero V., Garcia-Sanchez P., Honrado C., Ramos A., Morgan H. (2019). AC electrokinetic biased deterministic lateral displacement for tunable particle separation. Lab Chip.

[81] Calero V., Garcia-Sanchez P., Ramos A., Morgan H. (2020). Electrokinetic biased deterministic lateral displacement: Scaling analysis and simulations. J. Chromatogr. A.

[82] Akbarzadeh A., Rezaei-Sadabady R., Davaran S., Joo S.W., Zarghami N., Hanifehpour Y., Samiei M., Kouhi M., Nejati-Koshki K. (2013). Liposome: Classification, preparation, and applications. Nanoscale Res. Lett..

[83] Stremersch S., De Smedt S.C., Raemdonck K. (2016). Therapeutic and diagnostic applications of extracellular vesicles. J. Control. Release.

[84] Wiklander O.P.B., Brennan M.A., Lotvall J., Breakefield X.O., El Andaloussi S. (2019). Advances in therapeutic applications of extracellular vesicles. Sci. Transl. Med..

[85] Busatto S., Zendrini A., Radeghieri A., Paolini L., Romano M., Presta M., Bergese P. (2019). The nanostructured secretome. Biomater. Sci..

[86] Kalluri R., LeBleu V.S. (2020). The biology, function, and biomedical applications of exosomes. Science.

[87] Hochstetter A., Vernekar R., Austin R.H., Becker H., Beech J.P., Fedosov D.A., Gompper G., Kim S.-C., Smith J.T., Stolovitzky G. (2020). Deterministic lateral displacement: Challenges and perspectives. ACS Nano.

[88] Xia Y., Whitesides G.M. (1998). Soft lithography. Angew. Chem. Int. Ed. Engl..

[89] Sbalzarini I.F., Koumoutsakos P. (2005). Feature point tracking and trajectory analysis for video imaging in cell biology. J. Struct. Biol..

[90] Hunter R.J., Ottewill R.H., Rowell R.L. (1981). Zeta Potential in Colloid Science: Principles and Applications.

[91] Russel W.B., Russel W.B., Saville D.A., Schowalter W.R. (1991). Colloidal Dispersions.

[92] Bazant M.Z., Li D. (2008). Nonlinear Electrokinetic Phenomena. Encyclopedia of Microfluidics and Nanofluidics.

[93] Green N.G., Ramos A., Gonzalez A., Morgan H., Castellanos A. (2000). Fluid flow induced by nonuniform ac electric fields in electrolytes on microelectrodes. I. Experimental measurements. Phys. Rev. E Stat. Phys. Plasmas Fluids Relat. Interdiscip. Top..

[94] Gonzalez A., Ramos A., Green N.G., Castellanos A., Morgan H. (2000). Fluid flow induced by nonuniform ac electric fields in electrolytes on microelectrodes. II. A linear double-layer analysis. Phys. Rev. E Stat. Phys. Plasmas Fluids Relat. Interdiscip. Top..

[95] Green N.G., Ramos A., Gonzalez A., Morgan H., Castellanos A. (2002). Fluid flow induced by nonuniform ac electric fields in electrolytes on microelectrodes. III. Observation of streamlines and numerical simulation. Phys. Rev. E Stat. Nonlin. Soft Matter. Phys..

[96] Ramos A., Morgan H., Green N.G., Castellanos A. (1999). AC electric-field-induced fluid flow in microelectrodes. J. Colloid Interface Sci..

[97] Bazant M.Z., Squires T.M. (2004). Induced-charge electrokinetic phenomena: Theory and microfluidic applications. Phys. Rev. Lett..

[98] Levitan J.A., Devasenathipathy S., Studer V., Ben Y.X., Thorsen T., Squires T.M., Bazant M.Z. (2005). Experimental observation of induced-charge electro-osmosis around a metal wire in a microchannel. Colloids Surf. A-Physicochem. Eng. Asp..

[99] Thamida S.K., Chang H.C. (2002). Nonlinear electrokinetic ejection and entrainment due to polarization at nearly insulated wedges. Phys. Fluids.

[100] Wang Q., Dingari N.N., Buie C.R. (2017). Nonlinear electrokinetic effects in insulator-based dielectrophoretic systems. Electrophoresis.

[101] Morgan H., Green N.G. (2003). AC Electrokinetics: Colloids and Nanoparticles.

[102] Arnold W.M., Schwan H.P., Zimmermann U. (1987). Surface conductance and other properties of latex-particles measured by electrorotation. J. Phys. Chem..

[103] Ermolina I., Morgan H. (2005). The electrokinetic properties of latex particles: Comparison of electrophoresis and dielectrophoresis. J. Colloid Interface Sci..

