# Supplementary material for: Charge-Based Separation of Micro- and Nanoparticles
Source: Micromachines (Basel). 2020 Nov 18;11(11):1014. doi: 10.3390/mi11111014 (PMC7702211; doi:10.3390/mi11111014)
Supplement: Supplementary file 1 [file micromachines-11-01014-s001.zip › Supplementary Information.docx]

Supplementary Information

Charged-based separation of micro- and nanoparticles

Bao D. Ho ^1^, Jason P. Beech ^1^ and Jonas O. Tegenfeldt ^1,^*

^1^ Division of Solid State Physics and NanoLund, Physics Department, Lund University, PO Box 118, 22100, Lund, Sweden; bao.hodang@gmail.com (B.D.H.); jason.beech@ftf.lth.se (J.P.B.)

***** Correspondence: jonas.tegenfeldt@ftf.lth.se; Tel.: +46 46 222 8063

1. Device fabrication

**Table S1.** Device fabrication steps.

| **Tasks** | **Steps** | **Vendors** |
| --- | --- | --- |
| Making SU-8 mold | 1. Design a photo mask using L-Edit 2016 | L-Edit 2016: Mentor Graphics Corp., Wilsonville, Oregon, United States |
|  | 1. Have the mask printed by a photo mask making company | Delta Mask, Enschede, The Netherlands |
|  | 1. Dispense SU-8 onto a 3-inch silicon wafer and spin at a well-defined speed, according to the manufacturer’s instructions. This defines the thickness of the SU-8 layer and consequently the depth of the molded PDMS device. | SU-8: MicroChem, Newton, MA, USA |
|  | 1. Expose the wafer to UV-light in a Karl Suss contact mask aligner, then develop the un-exposed SU-8. | Karl Suss MJB4 soft UV, Munich, Germany |
|  | 1. Coat the SU-8 mold with a monolayer of trichloro (1H,1H,2H,2H-perfluorooctyl) silane (PFOTS) by molecular vapor deposition to prevent PDMS from sticking to the mold over many tens of uses | PFOTS: Sigma Aldrich, Saint Louis, MO, USA |
| Making PDMS devices (soft lithography) | 1. Mix PDMS base and PDMS curing agent (ratio 10:1 w/w) and de-gas to remove air bubbles | PDMS: Sylgard 184, Dow Corning, Midland, MI, USA |
|  | 1. Pour PDMS onto the SU-8 mold, and cure in an oven at 80° C for 1 hour. |  |
|  | 1. Peel the PDMS slab off the mold, cut out the region containing the device, perforate the device at reservoir positions with a puncher (1 mm inner diameter) |  |
|  | 1. Treat the feature surface of the device and a PDMS-covered glass slide with oxygen plasma, and gently place the device on top of the glass slide. | ZEPTO, Diener Plasma-Surface Technology, Ebhausen, Germany |
|  | 1. Glue silicone tubes (5 mm outer, 3 mm inner) onto the device to form inlet and outlet reservoirs | Silicone tubes: 228-0707, VWR International LLC, Radnor, PA, USA  Glue: Elastosil A07, Wacker Chemie AG, Munich, Germany |
|  | 1. Cure the bonded devices in an oven at 120° C for 24 hours to bring the PDMS surface back to its native hydrophobic state [1], making it stable for electrokinetic experiments. Store the device for future use. |  |

2. Device design

The devices have well-defined flow-focused central input stream to make it possible to closely follow the particle trajectories. The devices were designed for analytical purposes, *i.e.* to quantify lateral displacement of different types and sizes of particles, without the need for collecting them in different outlet reservoirs, hence there is only one outlet reservoir (Figure S1). A pressure difference and a voltage are applied between the sample/buffer reservoirs and the outlet reservoir. There are 35 different devices arranged on a 3-inch wafer, with different gap widths *G* (2 µm - 25 μm) and periods *N* (5, 10, and 20) to give a wide range of critical diameters *D_C_*, from 0.66 µm to 16.2 μm. Each device has one critical diameter. However, only the ones used in this work are shown in Table S2.


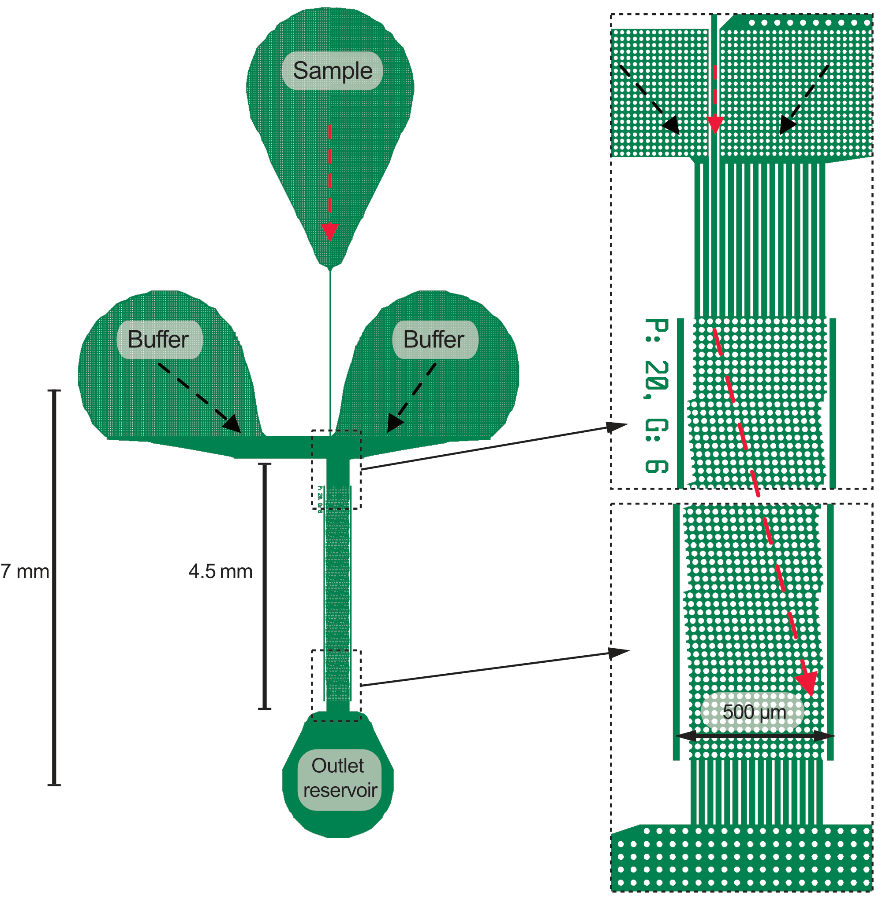


**Figure S1.** Overview of a typical device. The red arrows schematically represent the trajectory of displacing particles. The actual trajectory angles are given in Table S2. Drawings are to scale. Device #1-6 have similar DLD channel length, in the range 3.4-4.0 mm. The distance between buffer reservoirs and outlet reservoir, where the electrodes are placed, is around 7 mm. Device #7 is longer than the rest, with the array length of 9.5 mm, and electrode-to-electrode distance of 17 mm.

**Table S2.** Parameters of DLD devices used in this work. Critical diameters, *D_C_*, are nominal values given based on the geometry of the pillar array (Equation 1 in the main text).

| Device name | Gap [µm] | N | D_C_ [µm] | Deflection [θ] | Length  [mm] | Tested particles | Main text subsection |
| --- | --- | --- | --- | --- | --- | --- | --- |
| Device #1 | 2 | 20 | 0.66 | 2.86° | 3.6 | P.S. beads (160, 250 nm)  Liposomes (100-300 nm) | 3.3 |
| Device #2 | 5 | 10 | 2.32 | 5.71° | 4.0 | PS beads (2 µm) | 3.4 |
| Device #3 | 6 | 10 | 2.78 | 5.71° | 3.9 | PS beads (2 µm) | 3.2 |
| Device #4 | 8 | 10 | 3.71 | 5.71° | 3.9 | PS beads (1.1 µm) | 3.4 |
| Device #5 | 13 | 10 | 6.03 | 5.71° | 4.0 | PS beads (4.3 µm) | 3.1 |
| Device #6 | 13 | 5 | 8.41 | 11.3° | 3.4 | PS beads (6.3 µm) | 3.1 |
| Device #7 | 4 | 23 | 1.24 | 2.49° | 9.5 | P.S. beads (490 nm) | 3.3 |

3. Data analysis

3.1. Image processing

It is difficult to manually count particles sorted at high rate. Instead, the average image of an experimental image stack can be used to estimate the relative counts (Figure S2). Given the background noise is low, the concentration is not too high, the particles are uniform in size and brightness, and the particles travel with similar velocities, the integration of the averaged fluorescence intensity is a good basis for the estimation of the particle counts.


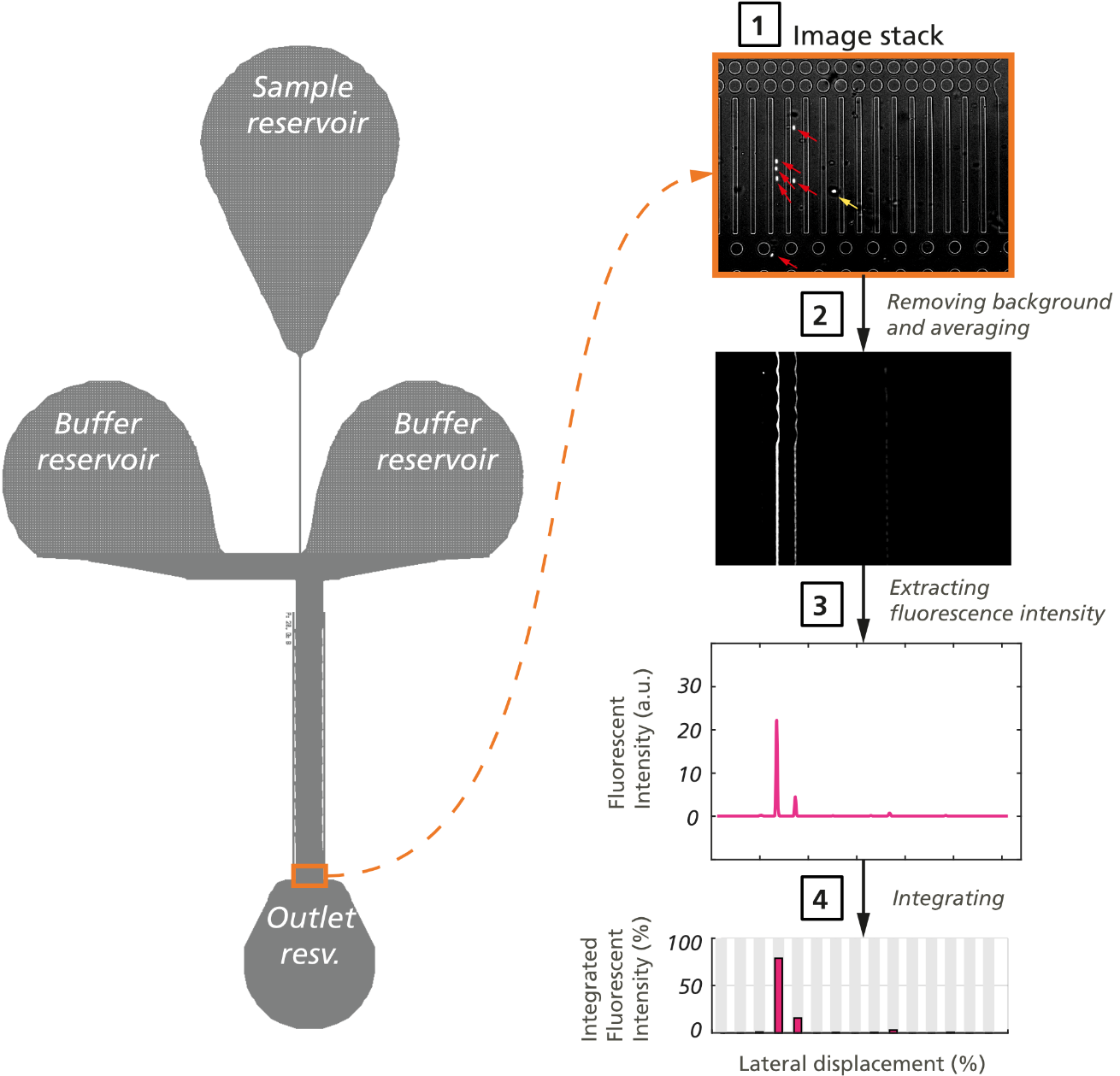


**Figure S2.** Image processing steps used to estimate relative particle counts based on fluorescence intensity. Step 1: Select region of interest (ROI). Note that the red arrows point to fluorescence polystyrene beads while the yellow arrow points to a defect in the device, which will be removed in the next step. Step 2: Remove background (by subtracting the average intensity of the whole stack from each image in the stack), make binary, and then average over all frames in the stack (for example, 3600 frames for 2 µm PS beads with different surface properties in Section 3.2 of the main text). Step 3: Collapse the data to the lateral dimension across the device by averaging along the longitudinal dimension. Step 4: Normalize and bin the data.

A comparison between manual counts and integrals of average fluorescence intensity of different types of beads, for different randomly chosen experiments, is shown in Figure S3. In general, integrals of fluorescence intensity agree well with manual counts, especially for the case of 2 µm sulphate particles #1 and #2. The slight mismatch in the case of the carboxyl #3 beads for example, is due to the variation in the size of the beads. Since the intensity is proportional to the amount of fluorophore, which is proportional to the volume of the beads, larger beads contribute more than smaller ones to the overall intensity and the method works less well for bead samples with large size distributions.


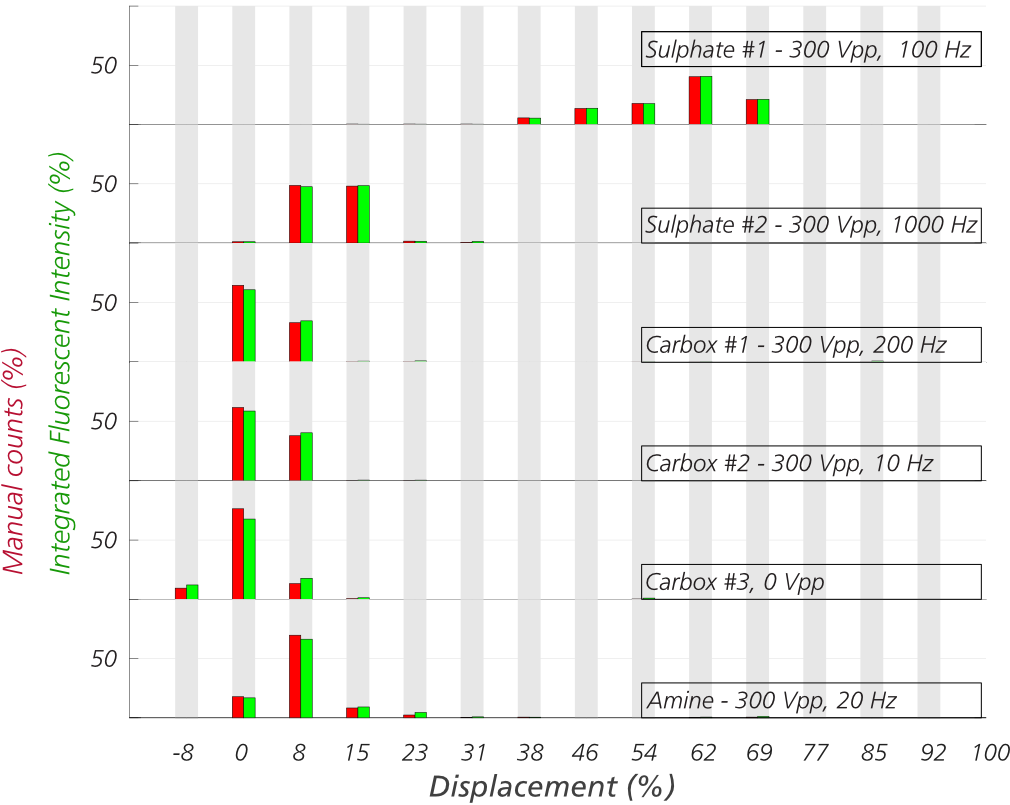


**Figure S3.** Relative amounts of 2 µm polystyrene particles as measured by manual counting and by integrated fluorescent intensity.

3.2. Data analysis

To study the effects of different running parameters (voltage, frequency, pressure, and medium conductivity) on particle displacement, the “particle counts *versus* displacement” data obtained from the image processing step above can be transformed to extract additional information (Figure S4). In the first step, multiple “counts *versus* displacement” plots at different voltages can be fitted into normal distributions and the means and standard deviations can be plotted into a “displacement *versus* voltages” graph to show how the displacement changes with the applied voltage. A similar “displacement *versus* frequency” can be plotted if the changing parameter is frequency instead of the voltage. In the second step, multiple “displacement *versus* voltages” plots at different pressures can be compressed into a “voltages *versus* pressures” plot, to show the relationship between the two. Here, each data point corresponds to the threshold voltage/pressure pair at which the particles are displaced halfway between zigzagging and displacing (50% displacement).


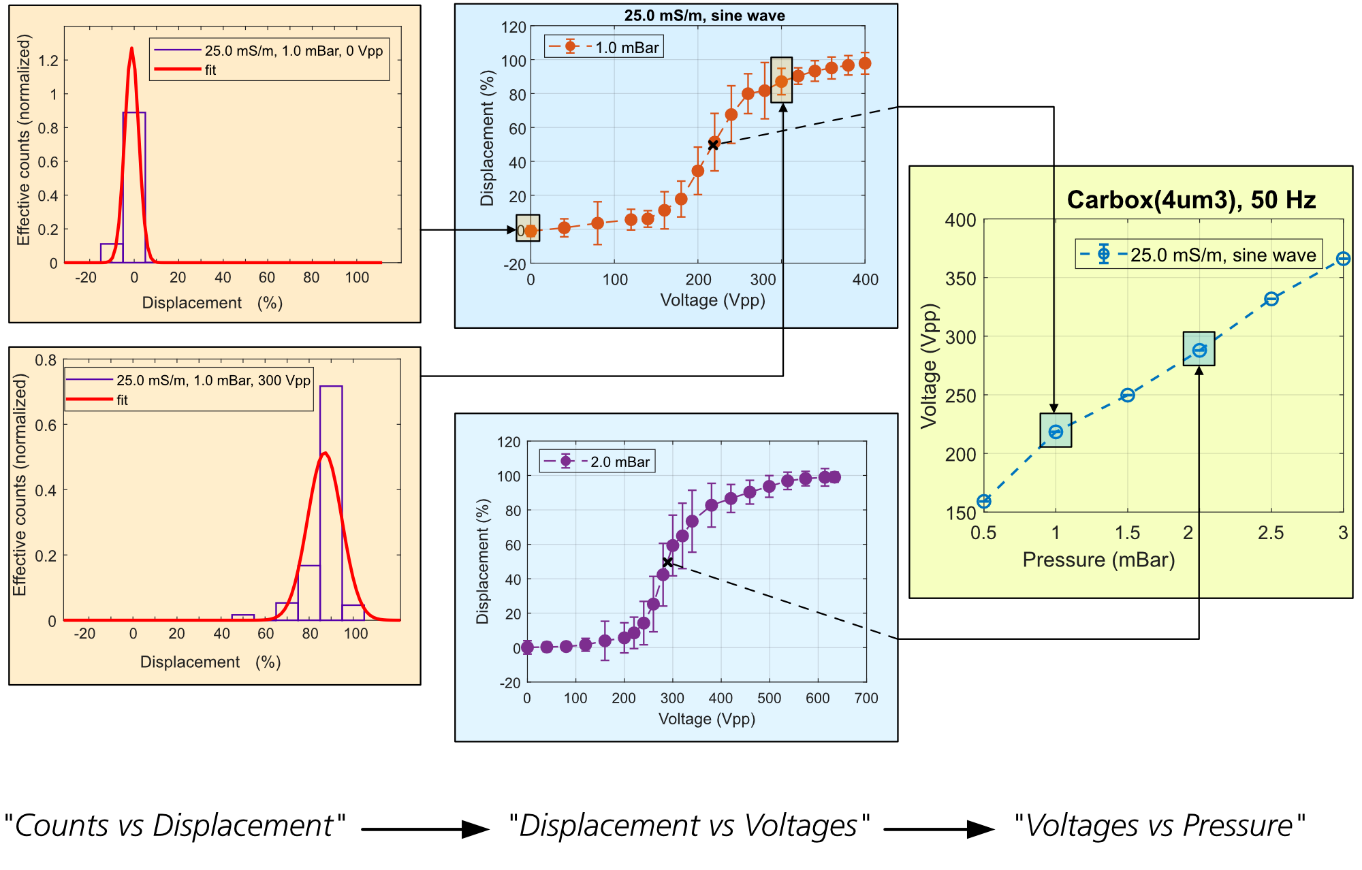


**Figure S4.** Summary of data transforming steps in order to investigate the effects of different running parameters on particle displacement.

4. Particles used for the experiments

The particles used in our work are listed in Table S3.

**Table S3.** Specifications of the particles used in this work. The media comprise KCl solutions with optionally added Pluronic® F127 0.1 % w/v to prevent sticking. The concentration of KCl was adjusted so that the electrical conductivity reached a desired value: 25 mS/m (1.7 mM KCl), 29 mS/m (2 mM KCl), and 500 mS/m (37 mM KCl). The conductivities were measured with a B-771 LAQUAtwin conductivity meter (resolution (range): 0.1 mS/m (0-20 mS/m), 1 mS/m (20 mS/m-200 mS/m), 10 mS/m (200 mS/m – 2000 mS/m)). The diameters of the polystyrene beads were reported by the vendors, whereas the sizes of the liposomes were measured with the Malvern Zetasizer NanoZS instrument using Dynamic Light Scattering. For the size measurements, the viscosity of the media is assumed to be that of pure water, since the viscosity change due to a small amount of KCl and Pluronic® is negligible [2,3]*.*


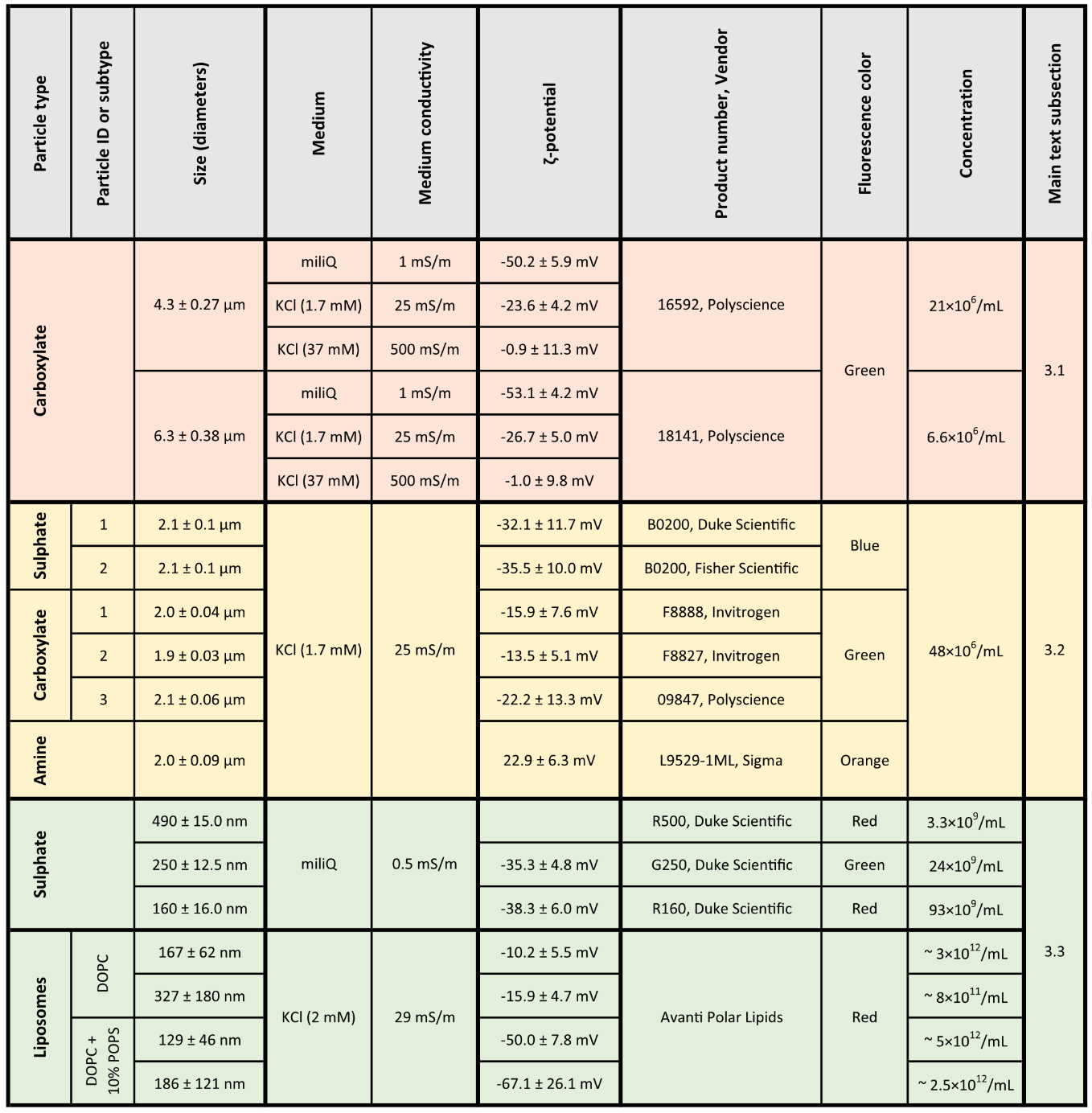


**4.1. Polystyrene microspheres**

Polystyrene (PS) microspheres with three types of surface modification were used: plain beads (having sulfate groups on their surface due to the nature of PS sphere production), carboxylate modified beads (having carboxylate groups grafted onto the surface), and amine beads (with grafted amine groups). Different size ranges of the beads were used (See Table S3), dependent on the experimental purposes.

- 1. **Polystyrene nanospheres and liposomes**

4.2.2. Optimization of polystyrene nanospheres sorting

Similar to other particles, 250 nm PS beads are displaced the most at $\sim50 Hz$ (Figure S5a). However, separation of 160 and 250 nm PS beads depends on their displacement contrast. For this purpose, $1000 Vpp/ 1kHz$ provide optimum conditions (Figure S5b). In Figure S 5b, we found that at 7.5 mBar, the separation is clearer and presented this data in the main text.


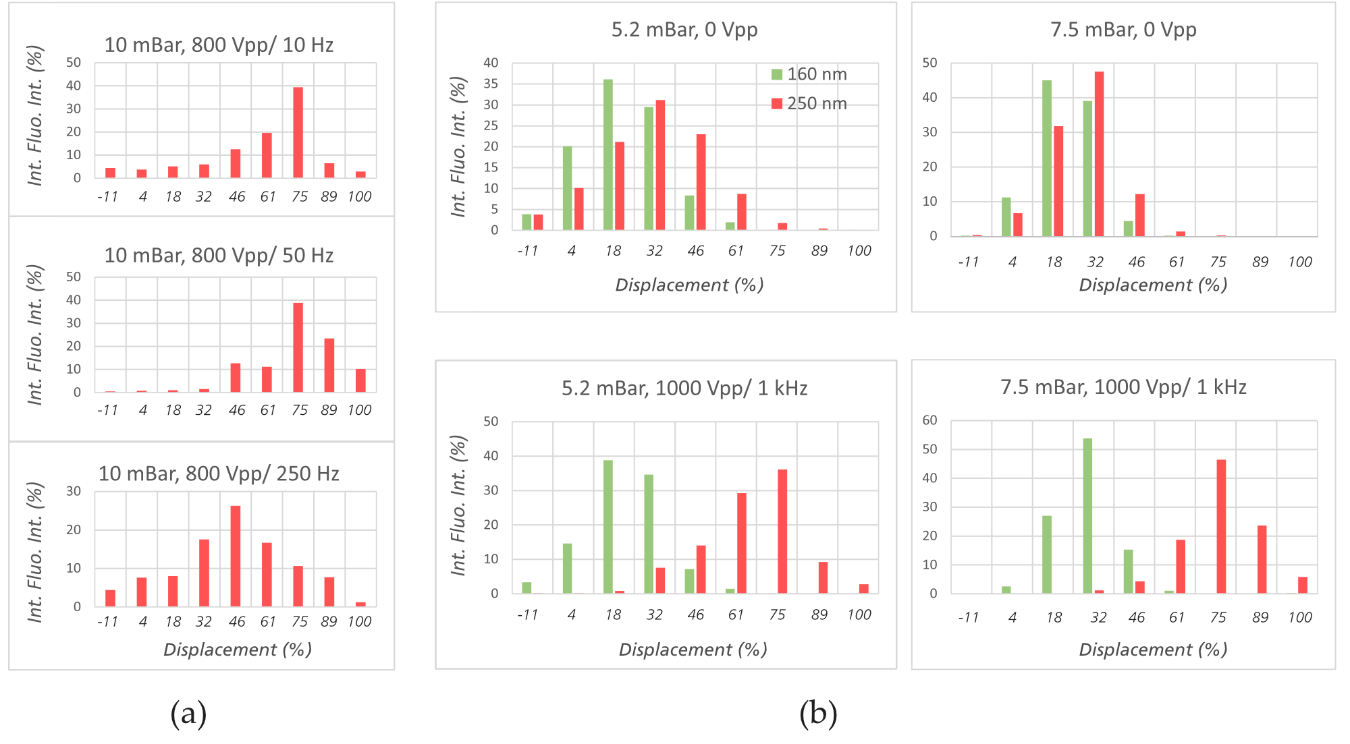


**Figure S5.** Optimization of nano PS bead sorting. a) 250 nm beads at different frequency, the particles are displaced the most at 50 Hz. b) Optimization of sorting between 160 and 250 nm beads with pressure as changing parameter. The medium conductivity is 0.5 mS/m.

4.2.3. Optimization of liposome sorting

In the main text (Section 3.3), we only show experimental data at 500 Vpp, where the best separation between DOPC/POPS 186 nm and the other liposomes can be observed. In Figure S6, more thorough data are presented, where the optimized parameter is the voltage. It is clear that by changing the voltage, different sorting profiles can be obtained. Interestingly at 1000 Vpp, large DOPC liposomes can be sorted from small DOPC liposomes. This also applies for DOPC/POPS liposomes.


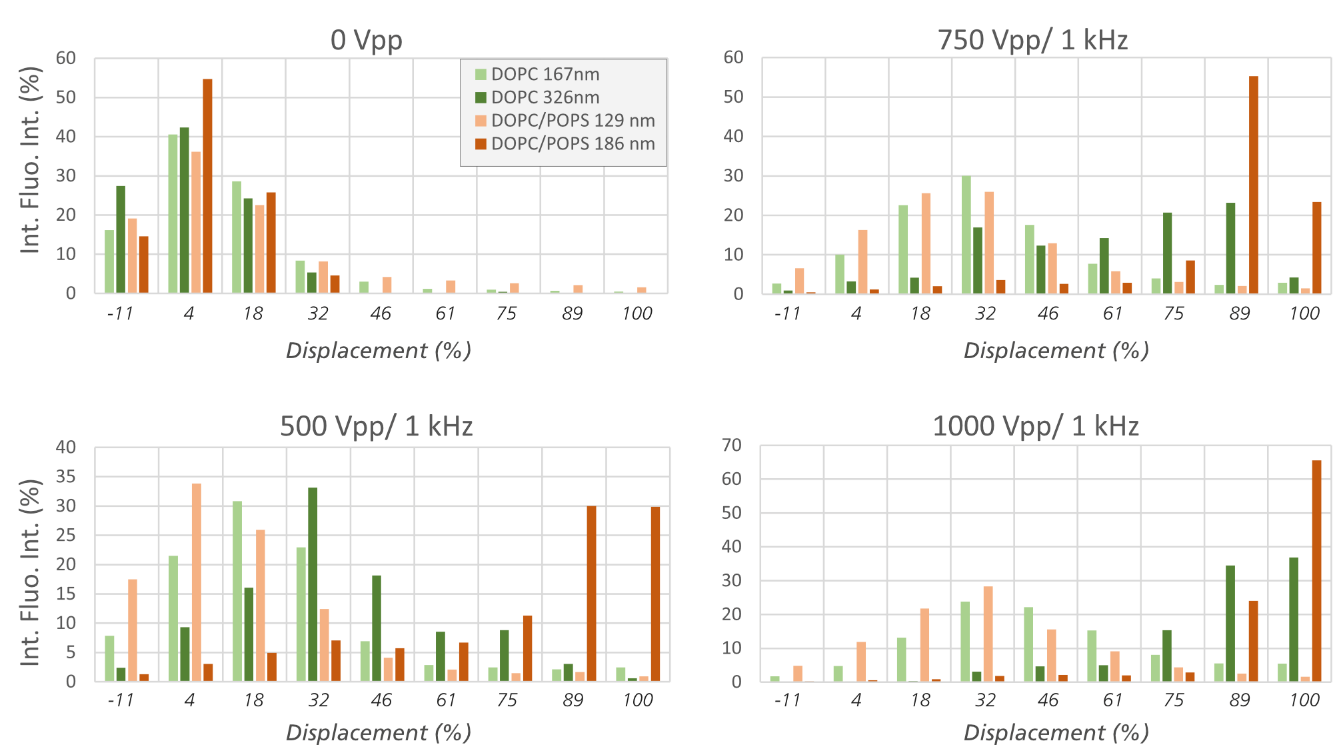


**Figure S6.** Optimization of liposome sorting, with voltage as the changing parameter. The frequency is 1 kHz and the medium is KCl 2mM, σ = 29mS/m, Pluronic® 127 is not needed.

5. Throughput and effects of diffusion

The throughputs of the devices are on the order of 1µL/hr and the Péclet numbers are much greater than unity. As an example, we calculate the throughput and the Péclet number of device #3 that was used to sort 2 µm PS beads.

At 10 mBar applied pressure difference across the device, the maximum velocity of the fluid is estimated at 870 µm/s (based on observed particle velocity in the middle of the gap between two posts). Assuming parabolic flow and noting that the depth (10 µm) >> gap (6 µm) between the posts, this corresponds to an average velocity of v_avg_ = 2/3 v_max_ = 580 µm/s. With 16 gaps across the width of the DLD channel the volumetric flow rate is estimated at:

$$Q = 16\times A_{cross_{section}}\times v_{avg}$$

$$Q= 16\times6 \mu m\times10 \mu m\times580 \frac{\mu m}{s}= 556 800 \mu m^{3}/s$$

$$Q \approx2.00\times{10}^{9} \frac{{\mu m}^{3}}{hour}= 2 \mu L/hour$$

We estimate the Péclet number as:

$$Pe \# = X^{2} / \sigma^{2}$$

Here the advective length scale *X* is the deflection length of the displacing particle. This is the length scale of interest since separation breaks down if diffusion is significant when compared to this length. The deflection of particles in displacement mode is (*L*: array length, *N*: array period):

$$X = \frac{L}{N}=\frac{3900 \mu m}{10}=390 \mu m$$

The time it takes to transport particles the whole length of the DLD array is simply:

$$t = L/v_{avg}=3900 \mu m / (580 \mu m/s) \approx6.7 s$$

Diffusion coefficient (assuming *2 µm* sphere in water):

$D = kT/6\pi\eta a= 4\times{10}^{-21}J/(6\times3.14\times0.93{\times10}^{-3}kg/(m.s)\times{1\times10}^{-6}m)$

$$D \approx2.3\times{10}^{-13} m^{2}/s = 0.23 \mu m^{2}/s$$

Diffusion length:

$$\sigma^{2}=2Dt=2\times0.23 \mu m^{2}/s* 6.7s$$

$$\sigma^{2}\approx3.1 \mu m^{2}$$

This gives us a Péclet number of $Pe\approx49 000$ for 2 µm particles in device #3. Similar calculations can be carried out for liposomes, and the results are shown in Table S4.

**Table S4.** Throughput and Péclet numbers of the experiments reported in the main text. Lengths of DLD arrays in both devices are within 3.6-3.9 mm. Polystyrene beads 2 µm (main text subsection 3.2), liposomes (main text subsection 3.3). In both cases, $Pe\gg1$.

| **Particle type** | **Particle diameter (µm)** | **Device** | **D_C_ (µm)** | **Pressure (mBar)** | **v_avg_ (µm/s)** | **Throughput (µL/h)** | **Diffusion length (µm)** | **Peclet number** |
| --- | --- | --- | --- | --- | --- | --- | --- | --- |
| Polystyrene beads | 2.00 | Dev. *#3* | 2.8 | 10 | 580 | 2.00 | 1.77 | 49×10^3^ |
| Liposomes | 0.19 | Dev. *#1* | 0.7 | 10 | 44 | 0.17 | 20.2 | 0.32×10^3^ |

6. Simple scaling relation between displacement, voltage, and pressure in eDLD

Although the geometry of the pillar array and the trajectory of the particles can be complicated, to a first approximation we can derive a simple scaling relation between the displacement, pressure, and voltage in eDLD under the electrokinetic wall force $F_{EW}$. In the x-direction, the electrokinetic force is balanced by the Stokes drag force. The particle travels with a velocity $v_{p_{x}}$ which gives rise to the electrokinetically enhanced displacement $\Delta x$ (Figure S7).


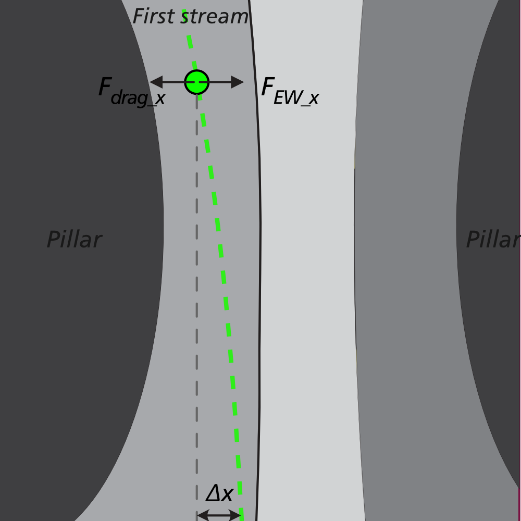


**Figure S7.** Simple sketch demonstrating the displacement caused by electrokinetic force.

In laminar flow, inertia of particles can be neglected. Therefore:

$$F_{{EW}_{x}}=F_{{drag}_{x}}=b\left| v_{fluid_{x}}-v_{p_{x}} \right|$$

Where *b* is the drag coefficient. The fluid velocity in x direction: $v_{fluid_{x}}=0$. Hence:

$$\begin{aligned} F_{{EW}_{x}}=bv_{p_{x}} \#\left( 1 \right) \end{aligned}$$

The displacement $\Delta x$ can be calculated as:

$$\begin{aligned} \Delta x=v_{p_{x}}t \#\left( 2 \right) \end{aligned}$$

Where *t* is the time the particle travels past a pillar in *y*-direction, which is inversely proportional to the particle velocity in the *y-*direction, and thus is inversely proportional to the applied pressure:

$$t\propto\frac{1}{v_{p_{y}}}$$

$$\begin{aligned} t\propto\frac{1}{P} \#\left( 3 \right) \end{aligned}$$

From Equation 1-3:

$$\Delta x\propto\frac{v_{p_{x}}}{P}=\frac{F_{{EW}_{x}}}{bP}$$

$$\begin{aligned} \Delta x\propto\frac{F_{{EW}_{x}}}{P} \#\left( 4 \right) \end{aligned}$$

From Equation 4, if $F_{{EW}_{x}}\propto V$ then $\Delta x\propto\frac{V}{P}$ and if $F_{{EW}_{x}}\propto V^{2}$ then $\Delta x\propto\frac{V^{2}}{P}$.

7. Fundamental of electrokinetics

**Table S5.** Important equations of electrokinetics. Symbols: $\sigma_{0}$ (surface charge), $\varepsilon$ (medium permittivity),$\kappa$ (inverse of Debye length), $\phi_{0}$ (surface potential), $k_{B}$ (Boltzmann constant), $T$ (absolute temperature), $q$ (proton charge), $y$ (distance from the surface), $\zeta$ (zeta potential), $t$ (thickness of the immobilized liquid layer at the wall), $n_{0}$ (number density of ions in the bulk), $c$ (molar concentration of ions), $pK_{a}$ (acid dissociation constant).

| **Equation name** | **Equation** | **Implication** |
| --- | --- | --- |
| 1. Debye length   (mono symmetrical electrolyte) | $\kappa^{-1}=\sqrt{\frac{\varepsilon k_{B}T}{2q^{2}n_{0}}}$ |  |
| 1. Debye length in meter   (as a function of ion molar concentration) | $\kappa^{-1}=1.764\times{10}^{-11}\sqrt{\frac{T}{c}}$ | Debye length decreases with ionic strength |
| 1. Grahame equation [4]   (for thin double layer, mono symmetrical electrolyte, at 25⁰C) | $\sigma_{0}=0.1174\sqrt{c}\times sinh(19.46\phi_{0})$ | Surface charge increases with surface potential |
| 1. Electrokinetic charge in C/m^3^ [5] (for thin double layer, mono symmetrical electrolyte, at 25⁰C) | $\sigma_{e}=0.1174\sqrt{c}\times sinh(19.46\zeta)$ | Electrokinetic charge increases with zeta potential |
| 1. Gouy-Chapman theory   (for thin double layer, mono symmetrical electrolyte) | $\phi= \frac{4k_{B}T}{q}tanh\frac{q\phi_{0}}{4k_{B}T}e^{-\kappa y}$ |  |
| 1. Zeta potential   (based on Gouy-Chapman theory) | $\zeta= \frac{4k_{B}T}{q}tanh\frac{{q\phi}_{0}}{4k_{B}T}e^{-\kappa t}$ | - $\zeta$ increases with $\phi_{0}$, and thus from Eq. 3, with$\sigma_{0}$. ***Suspended in the same electrolyte medium, particles with higher surface charge will have higher (absolute) zeta potential.*** - To the first order approximation, $\phi_{0}$ and $t$ are independent of ion concentration ^1^. Therefore when varying ion concentration, from Eq. 2 and Eq. 6, $\zeta\propto e^{-56.7\times{10}^{9}\sqrt{\frac{c}{T}}t}$. ***This means a particle’s zeta potential decreases with the salt concentration of the medium it is suspended in.*** |
| 1. Henderson–Hasselbalch equation | $pH=$  $pK_{a}+log\frac{\left[ proton acceptor \right]}{\left[ proton donor \right]}$ | The surface charge $\sigma_{0}$ of a particle is dependent on pH of the medium, pK_a_ of the charge group, and the number of charge groups per unit area of the particle surface. But it is independent of ionic strength of the solution. |

1. As an example, see Table II in [6].
2. Electric field simulations

We performed numerical simulations with COMSOL Multiphysics 5.4 to study the effects of period (N), gap (G), and post width (P) on electric field distribution in the device. The geometry includes N rows and four columns (Figure S8a) and a voltage was applied along the length of the geometry. The voltage was adjusted so that the average field along the device (without pillars) is 214 V/cm (the estimated average field obtained in device #5 if 300 Vpp was applied).

We investigated two scenarios: i) keeping Dc = 6.03 µm (constant) by changing N and G accordingly using Equation 1 in the main text, P was also kept unchanged; ii) keeping Dc = 6.03 µm (constant) by keeping G and N constant (G = 18.1 µm, N = 20) but changing P. In both cases, we examined the electric field at a constant distance, D_C_/2, away from the bumping pillar (Figure S8b). The idea is that among devices with different N, G, and P, can we select a specific combination of {N, G, P} that gives the strongest electric field at that chosen point, and as a results, produces the strongest electrokinetic effect to a particle traveling in the border between zigzag and displacement mode?

The results for case i) is presented in Figure S8c and case ii) in Figure S8d. The plots show that the field is not changing much (max 3%) when varying N (and G so that D_C_ is the same). However, increasing post width makes the field at the chosen point stronger by 35% (P = 5 µm *vs.* P = 60 µm).


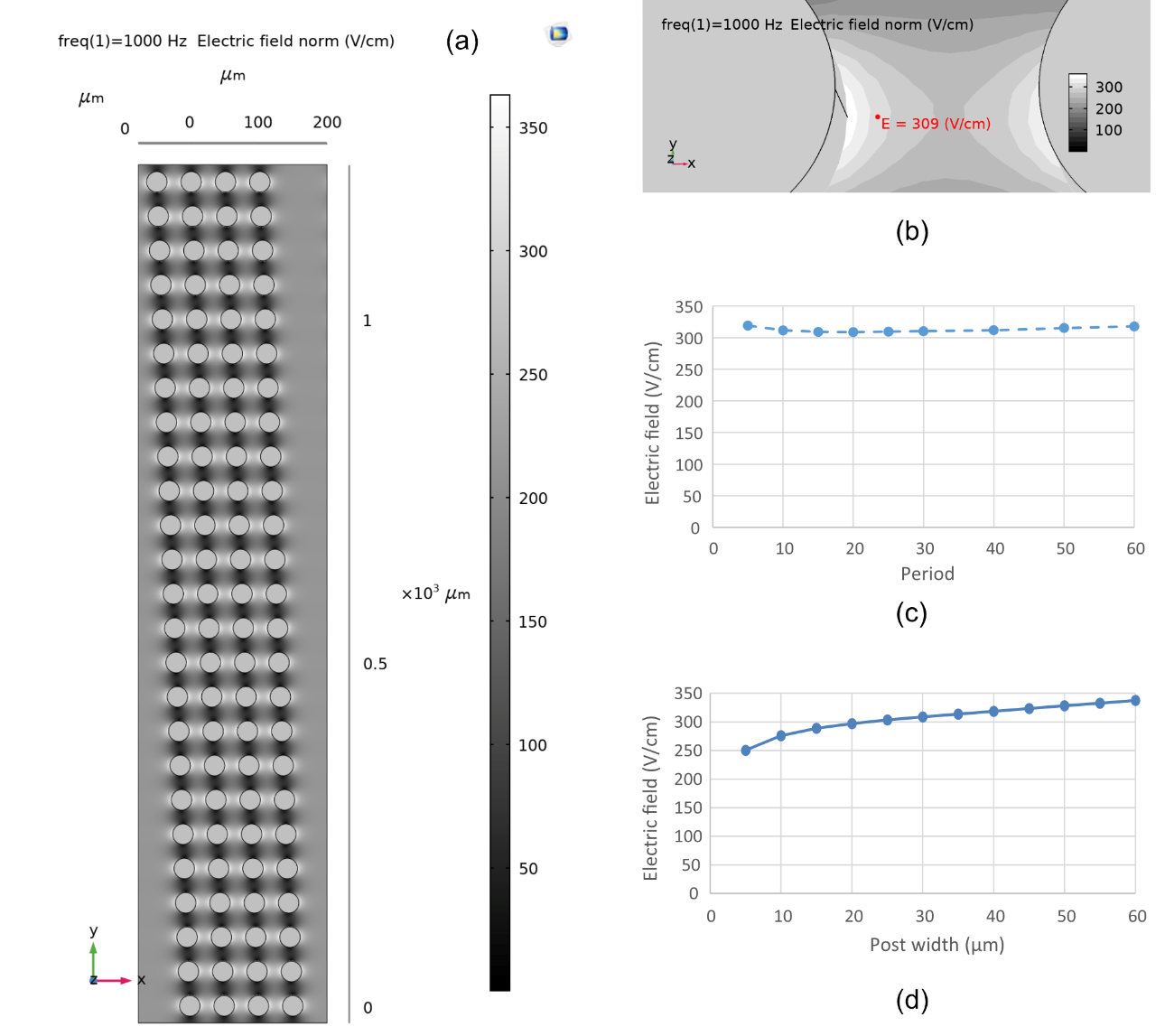


**Figure S8.** Electric field simulations of eDLD array with varying geometric parameters: N, G, and P. **a)** The geometry of the pillar array. **b)** The position where we examined the electric field: a distance of D_C_/2 away from the bumping pillar. **c)** Electric field at the chosen point in b) when varying N (and G so that D_C_ is the same). **d)** Electric field at the chosen point in b) when varying P (G, N, and D_C_ are the same).

References

1. Hung, L.-H.; Lee, A.P. Optimization of droplet generation by controlling PDMS surface hydrophobicity. In Proceedings of ASME 2004 International Mechanical Engineering Congress and Exposition; pp. 47-48.

2. Kestin, J.; Khalifa, H.E.; Correia, R.J. Tables of the dynamic and kinematic viscosity of aqueous KCl solutions in the temperature range 25–150 °C and the pressure range 0.1–35 MPa. *Journal of Physical and Chemical Reference Data* **1981**, *10*, 57-70, doi:10.1063/1.555640.

3. Kurumada, K.-i.; Robinson, B.H. Viscosity studies of pluronic F127 in aqueous solution. Berlin, Heidelberg; pp. 12-15.

4. Morgan, H.; Green, N.G. *AC Electrokinetics: Colloids and Nanoparticles*; Research Studies Press, Hertfordshire, UK, 2003.

5. Hunter, R.J.; Ottewill, R.H.; Rowell, R.L. *Zeta Potential in Colloid Science: Principles and Applications*; Academic Press: 1981.

6. Eversole, W.G.; Boardman, W.W. The Effect of Electrostatic Forces on Electrokinetic Potentials. *The Journal of Chemical Physics* **1941**, *9*, 798-801, doi:10.1063/1.1750846.
